# Investigating the transcriptional fingerprints of cocultured *Saccharomyces cerevisiae* and *Lachancea thermotolerans* in a model wine environment

**DOI:** 10.3389/fmicb.2025.1720597

**Published:** 2026-01-02

**Authors:** Justin Joseph Asmus, Rene Kathleen Naidoo-Blassoples, Roberto Pérez-Torrado, Florian F. Bauer

**Affiliations:** 1 Department of Viticulture and Oenology, South African Grape and Wine Research Institute, Stellenbosch University, Stellenbosch, South Africa; 2 Instituto de Agroquímica y Tecnología de los Alimentos, IATA-CSIC, Paterna, Spain

**Keywords:** coculture, *FIT2*, interactions, pooled analysis *Lachancea thermotolerans*, *Saccharomyces cerevisiae*

## Abstract

**Introduction:**

Wine fermentation is an evolutionarily relevant and relatively well described microbial ecosystem that was proposed as a model system to study mechanisms of interactions between wine yeast species. In this context, several studies have investigated phenotypic and molecular characteristics of yeast species when in two-species coculture, consisting of one strain of *S. cerevisiae* and a strain of another prevalent wine yeast species, including *L. thermotolerans* and *T. delbrueckii.*. Transcriptomic data generated in such studies have highlighted *S. cerevisiae* genes whose expression appeared to respond to the presence of other yeasts. However, these datasets diverge due to different growth conditions, differing inoculation strategies, the strains that were used and sampling time points.

**Methods:**

In this work, a pooled analysis was conducted to combine and integrate datasets generated from previous studies involving interaction between *S. cerevisiae* and *L. thermotolerans*. Thirty-nine samples from three studies generated on Illumina or Ion Torrent sequencing platforms were individually re-assessed using iDEP for normalization and differential expression analysis (|log_2_FC| > 0, FDR ≤ 0.05). Recurring trends in the form of a core set of differentially expressed genes were identified. Deletion mutants of these genes were evaluated in a semi-high throughput assay to identify genes whose activity would specifically impact growth and fermentation performance in cocultures, and one *S. cerevisiae* gene, *FIT2*, whose deletion mutants consistently showed diverging phenotypes when in coculture, was further analyzed.

**Results and discussion:**

The results highlight pathways and genes consistently enriched in all studies, including copper ion import, transition metal and iron ion transport, cell wall mannoproteins and biogenesis as well as methionine and sulfur biosynthesis. Interestingly, *FIT2* deletion in the original wine yeast wildtype strain (VIN13Δ*fit2*) showed opposite, but still interaction specific, phenotypes when compared with the laboratory strains of the Euroscarf deletion library. Considering the evolutionary context of these strains and likely differences in cell wall mannoprotein composition, these data emphasize the challenges of gene annotation in an ecosystem relevant context. The findings reinforce observations from previous research, suggesting that *FIT2* has a significant role in modulating interactions between species and highlighting specific DEGs from pathways that require further investigation in future coculture studies.

## Introduction

The use of mixed species cultures in wine production has become prevalent in recent years and usually involves the inoculation of grape must with *Saccharomyces cerevisiae* and at least one non-*Saccharomyces* yeast (Chasseriaud et al., 2023; Ciani et al., 2010; Comitini et al., 2021; Comitini et al., 2011; Conacher et al., 2021; Conacher et al., 2020; Milanovic et al., 2012). Indeed, it has been widely accepted that the addition of other yeast may contribute to desirable wine properties such as improved aromatic profiles, higher lactic acid content, reduced alcohol levels, reduction in volatile acidity and increased wine polysaccharide and glycerol concentrations (Bagheri et al., 2018; Binati et al., 2020; Comitini et al., 2011; Hranilovic et al., 2018). It was also highlighted that the interactions between yeast species during fermentation significantly impacted on the performances and metabolism of these yeasts within the wine microbial ecosystem, and data suggest yeast interaction-specific molecular responses.

Most wine fermentations are characterized by dominance of *S. cerevisiae* in the latter stages of the process. This dominance is linked to several adaptations to growth in a high-sugar environment, including a high ethanol tolerance combined with an ability to grow and ferment in the near, but not total, absence of oxygen (Gao and Fleet, 1988; Stanley et al., 2010; Williams et al., 2015)(Alonso-del-Real et al., 2019; Bagheri et al., 2018; Shekhawat et al., 2017). Other prominent wine yeasts, such as *Lachancea thermotolerans* and *Torulaspora delbrueckii*, also produce ethanol and grow well under oxygen-limited conditions in coculture with *S. cerevisiae*, but they are inhibited and outcompeted by *S. cerevisiae* as fermentation conditions shift in favor of this yeast (Shekhawat et al., 2017). Several studies investigating these and other traits have generated phenotypic data for inter and intra-species interactions, however the underlying molecular mechanisms remain poorly characterized (Brou et al., 2018; Comitini et al., 2021; Conacher et al., 2022; Contreras-Ruiz et al., 2025; Englezos et al., 2019; Luyt et al., 2024; Mejias-Ortiz et al., 2025; Wang et al., 2024).

Transcriptomic profiling of *S. cerevisiae* and other yeasts was primarily applied in single species cultures and has provided large data sets characterizing the genetic regulation of many aspects of yeast cellular metabolism and physiology (Minebois et al., 2021; Rossouw et al., 2009; Shekhawat et al., 2020). However, these data represent an experimental framework that is not applicable within an evolutionarily relevant context since yeast cells in the natural environment would never be found in monocultures. Indeed, yeast always interact and compete with several other species (Acosta-García et al., 2023; Alonso-del-Real et al., 2019; Contreras-Ruiz et al., 2025). Few studies have tried to apply omics methodologies within this evolutionary context partly due to the complexity of such systems. To gain insight into evolutionary relevant gene regulation in multispecies systems, omic analysis has been applied to systems with reduced complexity, e.g., two-species cocultures, with the aim of defining interaction-related transcriptomic signatures in mixed species fermentation (Fu et al., 2024; Luyt et al., 2024; Shekhawat et al., 2019).

While less complex than natural ecosystems, studying two-species cocultures presents several challenges. In these systems, cells of two species continuously respond to environmental changes, while simultaneously responding to the presence of other yeasts in their ecosystems (Conacher et al., 2021, 2022; Conacher et al., 2019). To identify gene expression patterns that would be specifically related to yeast interactions in these conditions is challenging and highly context-dependent (Conacher et al., 2024). The data indeed show that minor differences in the initial conditions of the cocultures linked to inoculation strategies have a significant impact on the phenotypic responses and the overall development of the cocultures. The phenotypic and molecular effects of interactions in cocultures depend on relative cell concentrations and the combined metabolic activities of both species which are rapidly and continuously changing throughout the coculture.

Conacher et al. (2024) highlighted that even small differences in pre-culturing, including the growth phases at which cells were harvested prior to inoculation and inoculation ratios of species in mixed fermentations had a significant impact on ecosystem development. Mixed fermentations with several species, including *L. thermotolerans*, *S. cerevisiae*, *T. delbrueckii* and *Wickerhamomyces anomalus* revealed that the preculture medium of the inoculums was a significant factor in the temporal succession of each yeast species throughout fermentation. While inoculation dosage used for each yeast in mixed culture pairings was found to be a determining factor in non-*Saccharomyces* succession patterns during fermentation. Changes in these parameters, however, have minimal impacts on the growth of single-species cultures.

Several other studies have specifically focused on cocultures between *S. cerevisiae* and *L. thermotolerans*. Shekhawat et al. (2019) followed a unique experimental strategy to reduce noise introduced by environmental factors, or by differences in inoculation ratios between yeast cultures, by comparing transcriptomic changes in interactions between *S. cerevisiae* and *L. thermotolerans* in steady state populations of mono- and mixed cultures of these yeasts under different oxygen levels in a continuous fermentation bioreactor system. Luyt et al. (2024) focused on responses related to physical cell–cell contact between these yeasts. Each of these studies identified specific genes that appeared to respond to the presence of a second species, suggesting that some of these genes may have specific molecular roles in responding to competing species.

With all the aforementioned in mind, we carried out a meta-transcriptomic analysis using sequencing datasets from previous studies (Conacher et al., 2022; Luyt et al., 2024; Shekhawat et al., 2019) conducted in our research group that consistently made use of the same *L. thermotolerans* strain. These studies used different growth conditions to evaluate the impact of coculturing when compared to single cultures. The aim was to identify any gene expression signature that would apply to all these studies, independently of the specific conditions. A subset of common DEGs identified in *S. cerevisiae* by comparative analysis of transcriptomes were screened for functional relevance within a species interaction context. For this phenotypic evaluation, a semi-high throughput screen was designed, monitoring growth of *S. cerevisiae* deletion mutants from the Euroscarf collection when cocultured with a fluorescently labeled (BFP) *L. thermotolerans* strain. Pairings that generated interesting phenotypic profiles were further analyzed.

The wildtypes possessing *FIT2* gene, encoding a cell wall mannoprotein of unknown function, consistently demonstrated significant coculture-dependent transcriptional responses, while the deletion strains consistently displayed a changed interaction pattern in the context of the screen. For this reason, the gene was also deleted in the original, evolutionarily more relevant wine yeast strain that had been used to generate the transcriptomic data. This new deletion strain also showed interaction-specific phenotypes that however differed from those displayed by the laboratory strain. Considering that *FIT2* encodes a cell wall mannoprotein, and that laboratory and wine yeast strains show significant differences in general cell wall properties and mannoprotein composition (Govender et al., 2010), differences in cell wall-dependent interaction phenotypes are not surprising. The data suggest that *FIT2* plays a significant role in yeast species interactions. The data also support strain-specific responses to coculture, highlighting the complexity of yeast ecosystem interactions.

## Materials and methods

### Yeast strains and culture media

The information regarding yeast strains that were used to generate transcriptomic datasets analyzed in our study, as well as the deletion mutant library reference strains can be found in Table 1. In this study, yeasts were aseptically revived from glycerol stocks (25% (w/vol)) stored at −80 °C whereafter these cultures were plated onto Wallerstein Laboratory (WL) nutrient agar (Sigma-Aldrich, Saint Louis, Missouri, United States) and incubated at 30 °C for 72 h before being used for further experimentation. Preculturing in each case, involved aseptically inoculating single colonies of each yeast strain from WL agar into 5 mL yeast peptone dextrose (YPD) broth test tubes (Sigma-Aldrich). Yeast cultures were incubated at 30 °C on rotary shaker wheel at 40 revolutions per minute (rpm) (Stuart SB3, Thermo Fisher Scientific, Waltham, MA, United States) for approximately 18- to 20 h until early stationary phase. Other media that were used for culturing yeast strains included a modified Synthetic Grape Must (SGM) from the medium that was described by Luyt et al. (2021), as well as an adaptation of SGM described by Viana et al. (2014) referred to by these authors as ISA-SGM (Instituto Superior de Agronomia – Synthetic Grape Must). The SGM and ISA-SGM prepared in our study were adjusted to pH 3.5 with 10 M KOH and both consisted of 200 g/L sugar (100 g/L glucose and 100 g/L fructose), which differed from concentrations used by Luyt and co-workers (2021) and Viana et al. (2014). Other components remained unchanged and included 2.5 g/L KH tartrate, 3 g/L ʟ-malic acid, 0.2 g/L citric acid, 1.14 g/L K_2_HPO_4_, 1.23 g/L MgSO_4_·7H_2_O, 0.44 g/L CaCl_2_·2H_2_O, 0.46 g/L NH_4_Cl and anaerobic factors (10 mg/L ergosterol and 0.5 mL/L tween), as well as amino acids, vitamins and trace elements (Supplementary Table S1). It should be noted that ISA-SGM contained additional components, e.g., uracil (120 mg/L), and differed in final concentrations of certain amino acids, including methionine (80 mg/L), leucine (400 mg/L) and histidine (100 mg/L).

**Table 1:** Yeast strains that were used in the current work, their origins and previous research involving these organisms.

Yeast	Strain	Origin	Study
*S. cerevisiae*	VIN13 mCherry (*TDH3-MCHERRY KANMX*)	Anchor Yeast, Cape Town, South Africa	Conacher et al. (2022) and Conacher et al. (2020)
	cross evolution-285	Lallemand SAS, Blagnac, France	Shekhawat et al. (2019)
	Lalvin EC1118	Lallemand Inc., Montreal, QC, Canada	Luyt et al. (2024)
	BY4741 (MATa *his3Δ1 leu2Δ0 met15Δ0 ura3Δ0*)	EUROSCARF, Scientific Research and Development GmbH, Köhlerweg, Oberursel, Germany	Baker Brachmann et al. (1998)
	BY4742 (MATα *his3Δ1 leu2Δ0 lys2Δ0 ura3Δ0*)		
*L. thermotolerans*	IWBT Y1240 BFP (*KLTH0G15730-GFP NATMX*)	South African Grape and Wine Research Institute (SAGWRI), Stellenbosch University, South Africa	Conacher et al. (2022) and Conacher et al. (2020)

### Pooled analysis of transcriptomic datasets for *Saccharomyces cerevisiae* and *Lachancea thermotolerans* cocultures

For this analysis, transcriptomic datasets of *S. cerevisiae* and *L. thermotolerans* were re-analyzed and compared from three different studies to identify shared DEGs for the response of these yeasts to coculturing under various fermentation conditions used in each study (Conacher et al., 2022; Luyt et al., 2024; Shekhawat et al., 2019). Each dataset was analyzed according to a bioinformatic pipeline that made use of freely available software.

### Pre-processing of datasets

**Figure 1:**
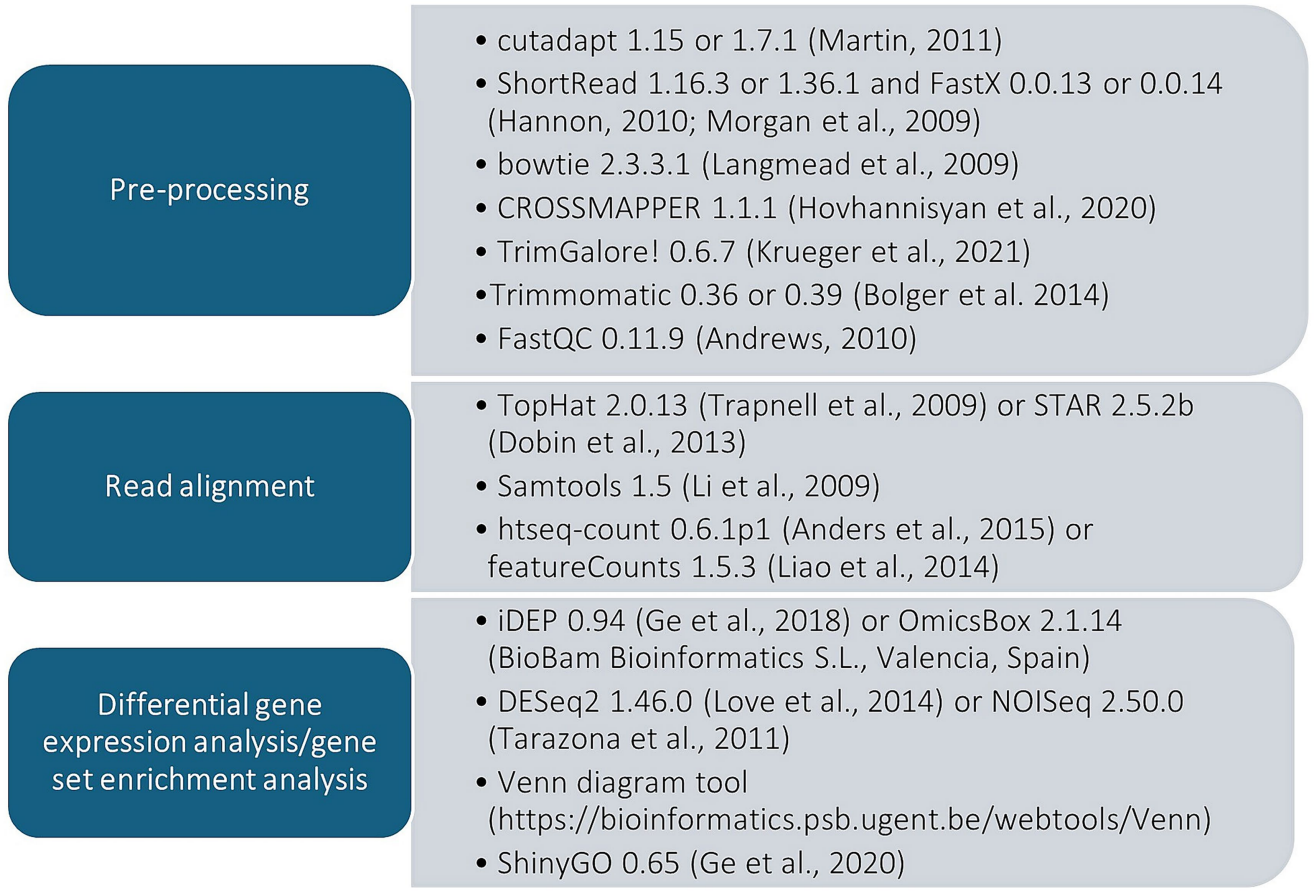
Summary of the pipelines followed for the transcriptomic datasets that were analyzed in this study.

**Figure 2:**
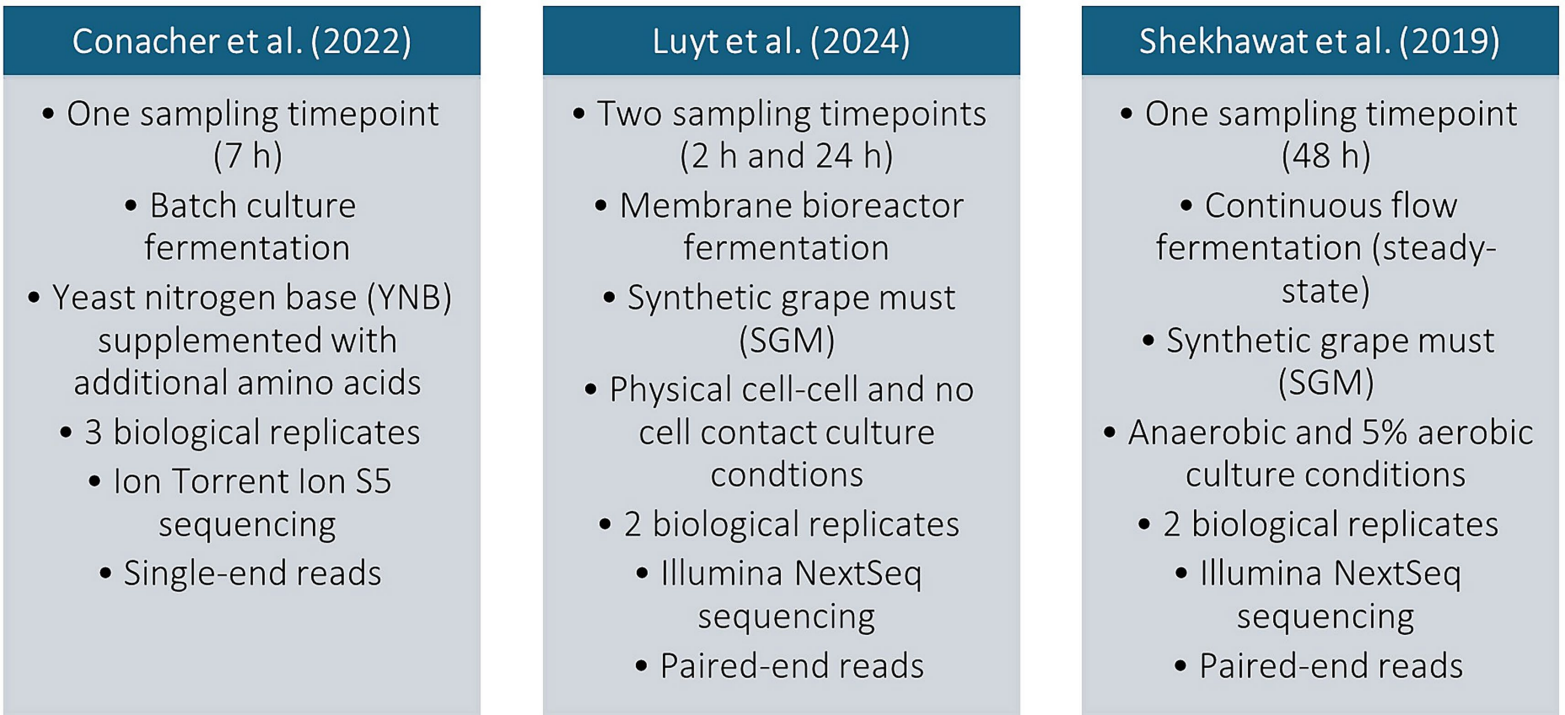
Different experimental conditions and sequencing strategies that were used for cocultures involving *S. cerevisiae* and *L. thermotolerans* in previous studies whose transcriptional datasets were re-analyzed in the current work.

Similar pre-processing and alignment steps were followed for the datasets and are summarized (Figure 1) along with descriptions of how conditions differed between the studies that were investigated (Figure 2). For the datasets of Shekhawat et al. (2019) and Luyt et al. (2024) the raw read counts data was already generated and available for re-analysis of differentially expressed genes. The specifics of the data pre-processing steps in the respective studies included trimming of reads with low quality ends (Q < 20) and subsequent removal of reads shorter than 35 base pairs (< 35 bp) using FastX 0.0.13 or 0.0.14 (Hannon, 2010). Hereafter, adapters were trimmed only at the ends using cutadapt 1.15 or 1.7.1 (Martin, 2011) and resultant reads < 35 bp were once again removed. Then low-quality (Q < 25) reads, polyA-containing reads, as well as ambiguous (N-containing) and artifact reads were removed using ShortRead 1.16.3 or 1.36.1 (Morgan et al., 2009) and FastX. This was followed by the removal of broken pair reads and contaminants using Trimmomatic 0.36 or 0.39 (Bolger et al., 2014) and bowtie 2.3.3.1 (Langmead et al., 2009), after which processed reads were assessed for quality using ShortRead. Thereafter, read alignment was performed using either TopHat 2.0.13 (Trapnell et al., 2009) or STAR 2.5.2b (Dobin et al., 2013) mapping tools, respectively. Reads were aligned to a chimeric genome of *S. cerevisiae* and *L. thermotolerans*, based on the inconsequential rate of read crossmapping between these yeasts genomes (below 1%), which is supported by the output of CROSSMAPPER 1.1.1 (Hovhannisyan et al., 2020; Supplementary Figure S1). Non-primary mapping reads or those that had mapping qualities ≤ 20 were removed using samtools 1.5 (Li et al., 2009), which was also used to sort and index the aligned reads according to chromosomes. Reads that mapped to gene features were then counted in each case with either featureCounts 1.5.3 (Liao et al., 2014) or htseq-count 0.6.1p1 (Anders et al., 2015) respectively. The dataset of Conacher et al. (2022) was originally pre-processed using Partek Flow software (Illumina Way, San Diego, CA, United States), however raw data from sequencing was re-analyzed in a similar manner to the processes described for Luyt et al. (2024) and Shekhawat et al. (2019). This included conversion of *bam* files to *fastq* format using samtools, the removal of short reads and quality assessment after read removal with TrimGalore! 0.6.7 (Krueger et al., 2021) and FastQC 0.11.9 (Andrews, 2010) respectively. Read alignment was then performed using STAR and reads with low mapping qualities (≤ 20) were removed using samtools, which was again used for sorting and indexing aligned reads to chromosomes. Afterwards, read counts were generated for the sorted aligned reads using featureCounts.

### Differential expression analysis

The raw counts for each dataset were then analyzed for differentially expressed genes (DEGs) using iDEP 0.94 (Ge et al., 2018). This online graphical user interface (GUI) makes use of compiled source code of R programming language packages used for gene expression analysis, including DESeq2 1.46.0 (Love et al., 2014), which was used to analyze the datasets used in our study. In these analyses, threshold cutoff values used to identify DEGs included |log_2_FC| > 0 and FDR ≤ 0.05, with the Benjamini–Hochberg multiple-testing correction method to reduce the likelihood of identifying false positives. However, for the *S. cerevisiae* anaerobic condition (AN) dataset that lacked biological replicates in the work of Shekhawat et al. (2019), OmicsBox 2.1.14 (BioBam Bioinformatics S. L., Valencia, Spain) was used for pairwise differential gene expression analysis (without replicates) by NOISeq 2.50.0 (Tarazona et al., 2011). In this program, differential gene expression between samples is determined using parameters “D” (D = value of absolute difference between two samples), “M” (M = log_2_FC of the two conditions being tested) and probability (e.g., probability of differential expression for each gene obtained by comparing the M and D values of a given feature against the noise distribution). Genes with probabilities higher than the default threshold (0.9) were considered to be differentially expressed between conditions. Hereafter, DEGs from datasets that were analyzed were overlapped using an online Venn diagram tool^1^ and the UpsetR Shiny tool (Lex et al., 2014) which was performed for up- and downregulated genes from each analysis, respectively. The final gene lists in both cases were also filtered using the DEGs that were obtained from one of the conditions tested by Luyt et al. (2024), which included differential expression of genes when cells were restricted to strictly metabolic contact (physically separated). These gene lists were then further analyzed by conducting a gene set enrichment analysis using another online Shiny tool ShinyGO 0.65 (Ge et al., 2020) to determine gene ontology (GO) terms and pathways associated with our genes.

### Phenotypic coculture screening of *Saccharomyces cerevisiae* deletion mutant strains with *Lachancea thermotolerans*

After identifying possible interaction genes from previous coculture studies involving *S. cerevisiae* and *L. thermotolerans* in the pooled analysis, these results were used to select *S. cerevisiae* deletion mutant strains to screen in cocultures with *L. thermotolerans*. These cocultures were performed to determine the impact of the absence of these genes in *S. cerevisiae* on interaction responses between the yeasts. Representatives of the EUROSCARF gene deletion mutant library of *S. cerevisiae* wildtype (WT) strains BY4741 and BY4742 were used for screening (EUROSCARF, Scientific Research and Development GmbH, Köhlerweg, Oberursel, Germany).

These yeasts were cultured with blue fluorescent protein (BFP) labeled *L. thermotolerans* IWBT Y1240 in SGM and ISA-SGM, where phenotypic profiles of yeast populations were monitored by flow cytometry using either the CytoFLEX (Beckman Coulter Life Sciences, Indianapolis, Indiana, United States) or MACSQuant® Analyzer 10 flow cytometers (Miltenyi Biotec, Bergisch Gladbach, Germany). The flow cytometers were equipped with blue (488 nm) and violet (405 nm) lasers, where BFP fluorescence was measured on the PB450 channel (450/45 BP) and propidium iodide (PI; 1 μM; Invitrogen, Thermo-fisher, Waltham, MA, United States) served as a viability stain and was measured on the PC5.5 channel (690/50 BP). Other relevant settings that were used included: 30 μL/min flow rate, 10,000 events collected per sample, gains for different channels were FSC: 151, SSC: 46, FITC:25, ECD: 268, PC5.5: 50, PB450: 37. The events/s measured were kept below 1,000 and the abort rate was less than 1%. An example of the gating and compensation strategy that was used can be seen in Supplementary Figure S15, which included gates to identify singlet data, cell viability (e.g., Live and PI Dead gates) and positive or negative BFP signal (e.g., BFP or NF). Prior to screening, viability gates were determined by killing wildtype *S. cerevisiae* cells and *L. thermotolerans* in absolute ethanol (Merck, Rahway, New Jersey, United States). Dead cells were positive for red fluorescence, while BFP positive cells separated from non-fluorescent *S. cerevisiae* cells (Supplementary Figure S15). For this experiment, yeasts were revived and precultured as mentioned earlier in YPD, after which cells were harvested (5,000 x *g*; 20 °C; 5 min) and resuspended in either SGM or ISA-SGM. Hereafter, cell concentrations of each yeast culture were adjusted by diluting cells in phosphate buffered saline (PBS; pH 7.2) with PI and enumerating cells by flow cytometry.

### Screening conditions and optimizations

Several optimizations were required for the screening, including, among others, confirming the reliability of the cell quantification method that was used (Supplementary Figure S4), assessing effects of preculture media and other conditions on growth of *S. cerevisiae* deletion mutants and WTs as well as determining which measurement of cell growth to use for differentiation in coculture experiments that were screened in coculture (Supplementary Figures S5–S7A,B), and preparation of ISA-SGM medium used to accommodate growth of *S. cerevisiae* auxotrophic strains (Supplementary Figure S8; Supplementary Table S1). In addition, different initial inoculation density ratios and sampling timepoints were tested for the coculture parings between *S. cerevisiae* and *L. thermotolerans* (Supplementary Figures S9, S10; Table 2). For the final screening conditions, pure and mixed micro-fermentations of *S. cerevisiae* WTs and mutants with *L. thermotolerans* were carried out in a final volume of 220 μL ISA-SGM in 96-well black/clear bottom microplates on a microplate shaker (Thermo Fisher Scientific) with a shaking speed of 600 rpm at between 22 to 25 °C after preculturing in YPD broth as mentioned.

**Table 2:** Parameters that were tested during optimization of phenotypic screening for yeast cocultures.

Yeast species in coculture	Inoculation ratios	Sampling timepoints
*S. cerevisiae* and *L. thermotolerans*	1:1 (1 × 10^6^ cells/mL:1 × 10^6^ cells/mL)	6-, 12-, 24- and 48 h
	2:1 (2 × 10^6^ cells/mL:1 × 10^6^ cells/mL)	
	2:0.5 (2 × 10^6^ cells/mL:0.5 × 10^6^ cells/mL)	

A final inoculation ratio density of 2:0.5 (2 × 10^6^ cells/mL:0.5 × 10^6^ cells/mL) was used for mono- and mixed cultures of *S. cerevisiae*: L*. thermotolerans*, which were randomly assigned to microplate wells and grown for 12 h before enumeration by flow cytometry. Monocultures for *L. thermotolerans* and *S. cerevisiae* WTs, as well as coculture pairings thereof, were included as controls and used as references for assessing the impact of gene deletions on coculture phenotypes. The results represent the mean viable cell numbers for three biological repeats with error bars representing the standard error of the mean in each case.

### Confirmation of phenotypic screening by CRISPR/Cas9 mediated whole gene deletion

To confirm the phenotypic profile that was observed for the pairings of the Δ*fit2* gene deletion mutant strains with *L. thermotolerans*, a CRISPR/Cas9 gene editing protocol based on the MoClo-Yeast Toolkit was followed to create a Δ*fit2* gene knock-out mutant of *S. cerevisiae* BY4741 and VIN13 WT strains (Lee et al., 2015; Novarina et al., 2022). Briefly, this involved designing single guide RNA (sgRNA) primers using CHOPCHOP v3 (Labun et al., 2019), which served as a target sequence for specific enzymatic cleavage of double-stranded DNA (dsDNA) inside the *FIT2* gene sequence within the genome, as well as primers for the repair DNA fragment that is required for gene deletion (Table 3). Thereafter, sgRNA oligos were phosphorylated and annealed *in vitro* to obtain ds-sgRNA, which was assembled with pWS175 plasmid^2^ according to Golden Gate method.

**Table 3:** Primer sequences for single guide RNA and repair DNA fragment that were synthesized for this study.

Primer name	Oligo sequence (5′- to −3′ orientation)
Forward primer sgRNA for *FIT2* knock-out	5’-CGGAAGCGAATTGAACGAGG-3’
Reverse primer sgRNA for *FIT2* knock-out	5’-CGCTGGTACTGGTTTGATGG-3’
Forward primer repair DNA fragment	5’-GGCAGAATTTTACGGTCCTTGTAAAAAAGTCTATCATAAAGCCATCACAAAACAATAATAGCTCGGTTTC-3’
Reverse primer repair DNA fragment	5’-TATTATTGTTATAAGTTATAAATATGCTATACACGATAACTAATAGCTTTTTTCTGGTTTGAAACCGAGC-3’
Forward primer for confirmation of *FIT2* gene deletion	5’-CGGAAGCGAATTGAACGAGG-3’
Reverse primer for confirmation of *FIT2* gene deletion	5’-CGCTGGTACTGGTTTGATGG-3’

The product was then transformed into *Escherichia coli* (NZYα) competent cells, after which plasmid extraction was performed on a positive transformant, while the repair fragment DNA was amplified by “no-template” PCR and purified for yeast transformations. The constructed plasmid and repair fragment were then transformed into competent cells of BY4741 and VIN13 according to the lithium acetate (LiAc)/SS carrier DNA/ PEG method described by Gietz and Schiestl (2007). To confirm success of CRISPR-mediated whole gene deletion of *FIT2*, DNA extractions were performed on representative colonies of *S. cerevisiae* VIN13 and BY4741 transformants that were cultured on YPD agar containing Hygromycin B (200 mg/mL) according to the method described by Lõoke et al. (2011). Thereafter, PCR amplification was performed with Ex Taq DNA polymerase (Takara Bio Inc., Kasatsu, Shiga, Japan) using primers (Table 3) designed to target the genomic flanking regions up- and downstream of *FIT2* and determine the presence (control) or absence (successful transformants) of the gene using the following cycle conditions: 95 °C for 3 min; 35 cycles of 94 °C for 30 s, 58 °C for 30 s, 72 °C for 1 min; 72 °C for 10 min. Amplification products were visualized on a 1% (w/vol) agarose gel containing ethidium bromide (0.5 μg/mL) and further analyzed by Sanger sequencing using the same primers mentioned, followed by alignment of sequences using MUSCLE 3.8 (Edgar, 2004; Supplementary Figure S13; Supplementary Text S1).

### Statistical analysis

The growth data for the phenotypic screening represents means of biological triplicates accompanied by standard error of the mean, unless otherwise stated. Descriptive statistics were performed on the data and normality was determined by normal probability plots. The data were then subjected to Levene’s test for homogeneity of variance and ANOVA (type III) analysis with Fishers LSD post-hoc tests. These analyses were performed in STATISTICA software version 14.0.1.25 (TIBCO Software Inc., Santa Clara, CA, United States) and performed at the 5% significance level, where *p*-values < 0.05 were considered statistically significant. Significant differences in cell numbers (*p*-value < 0.05) are indicated by lower-case letters, which compared yeast cultures per condition tested, e.g., “monocultures,” “*S. cerevisiae* cocultures” and “*L. thermotolerans* cocultures.” Bars with the same letters were not significantly different (*p*-value > 0.05) from one another within the tested condition, while bars with different letters differed significantly (*p*-value < 0.05).

## Results

### In silico analyses of transcriptomic data for yeast cocultures

Transcriptomic datasets from three different studies involving cocultures of different wine isolates of *S. cerevisiae* (e.g., EC1118, Cross evolution-285 and VIN13) and the same *L. thermotolerans* strain (IWBT Y1240) were reanalyzed to identify genes that were differentially expressed in all conditions. These yeasts were cultured in direct cell–cell contact in each study, therefore the DEG list that was created specifically related to this interaction (Figure 2).

**Figure 3:**
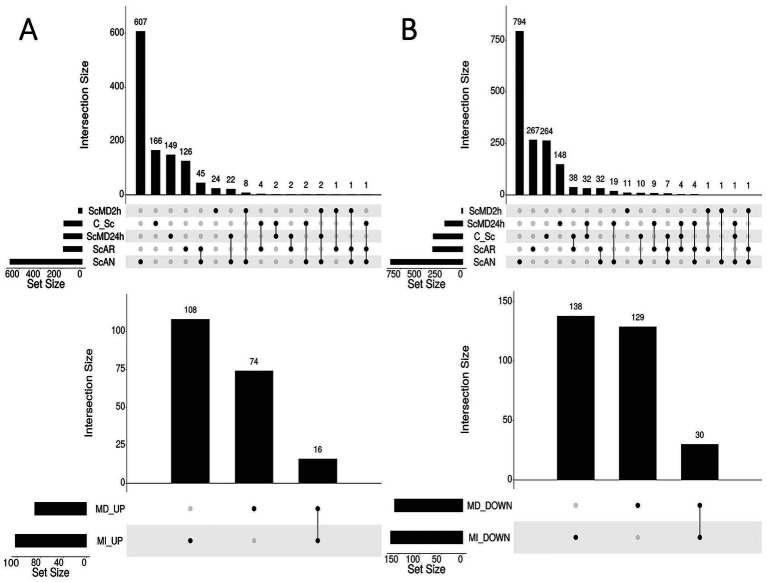
Upset plots demonstrating the process followed for the comparisons that were performed to identify **(A)** up- and **(B)** downregulated DEGs of interest in *S. cerevisiae* that were related to physical contact between this yeast and *L. thermotolerans* from re-analyzed coculture datasets. Independent nodes represent the total number of DEGs from each analyzed dataset, while connected nodes represent intersections (or shared DEGs) between these datasets and the number of these shared DEGs indicated above each comparison.

Upset plots were used to visualize intersections of DEGs of *S. cerevisiae* from each analysis, which was performed for up- and downregulated genes separately in each case (Figure 3). Comparisons included upregulated (3A) and downregulated (3B) genes from individually analyzed datasets of cocultures of *S. cerevisiae* and *L. thermotolerans*. One of these datasets included when these yeasts were cultivated in a continuous fermentation environment under aerobic (ScAR) and anaerobic (ScAN) conditions (Shekhawat et al., 2019). Data from yeasts that were cocultured in a membrane bioreactor under direct cell–cell contact for 2 h (ScMD2h) and 24 h (ScMD24h), as well as coculturing restricted to metabolic exchange (physically separated cells, e.g., ScMI2h and ScMI24h) were also assessed (Luyt et al., 2024). Additionally, transcriptional data of batch fermentations of these yeasts grown under aerated conditions for 7 h (C_Sc) were included (Conacher et al., 2022). The process followed for comparisons was as follows; C_Sc versus ScAR and ScAN, C_Sc versus ScMD2h and ScMD24h, ScAN versus ScMD2h and ScMD24h, and finally ScAR versus ScMD2h and ScMD24h. DEGs falling within the intersections of these comparisons were compiled into a preliminary list designated as mixed culture direct cell–cell contact (or MD). This process was repeated for the datasets generated from strictly metabolic exchange, where comparisons involving ScMD2h and ScMD24h were supplemented for ScMI2h and ScMI24h, which resulted in a list of DEGs resulting from indirect cell–cell contact (metabolite exchange) between yeasts in mixed culture (or MI). Preliminary DEG lists were compiled for both up- and downregulated gene datasets related to physical cell–cell contact between yeasts (e.g., MD_UP and MD_DOWN) and datasets related to indirect cell–cell contact (MI_UP and MI_DOWN). After duplicate DEGs were removed in each list, these datasets were compared (e.g., MI_UP versus MD_UP and MI_DOWN versus MD_DOWN) to remove genes in the MD_UP and MD_DOWN lists that overlapped with those found in MI_UP and MI_DOWN lists. Ultimately, we acquired genes only related to physical contact between cells, which reflected screening conditions.

The results focused on observations for *S. cerevisiae*, which yielded a total of 203 overlapping DEGs, consisting of 74 upregulated genes and 129 downregulated genes (Figure 3; Supplementary File S1). These genes were then further analyzed by enrichment analysis, which was summarized in Supplementary Table S2 and hierarchical clustering dendrograms displaying significant GO terms (Supplementary Figure S3A). Similar comparisons were performed for *L. thermotolerans* using Venn diagrams, which had 511 overlapping DEGs comprised of 264 and 247 genes from up- and downregulated gene comparisons, respectively, (Supplementary Figure S3B; Supplementary File S1).

Enrichment analyses were performed on the resultant gene lists (Supplementary Table S3; Supplementary Figure S4). Significant pathways (*p-value* < 0.05) with the highest fold-enrichment and false discovery rate (FDR) enrichment scores were included. For *S. cerevisiae*, genes in the upregulated DEG list were associated with copper ion import, transition metal and iron ion transport, respectively, while the downregulated DEG list genes were enriched in methionine and sulfur biosynthesis (Supplementary Table S2). For *L. thermotolerans*, highly enriched pathways contained genes related to ergosterol, phytosteroid and cellular alcohol biosynthetic processes for the upregulated DEGs that were analyzed, while the downregulated DEGs showed enrichment for *de novo* inosine monophosphate (IMP) biosynthetic and metabolic processes (Supplementary Table S3; Supplementary Figure S3B). Recently, fatty acid degradation and biosynthesis, among other metabolic processes including nitrogen metabolism, were found to be enriched in subpopulations of wild and domesticated strains of this yeast (Vicente et al., 2025a). These pathways were used to distinguish between yeast strains with different environmental origins and they were shown to be distinct between different groups. To further investigate the possible phenotypic impact these genes could have on coculture dynamics, we performed screenings with *L. thermotolerans* grown together with deletion mutants of the identified *S. cerevisiae* genes.

### Yeast coculture screening

To determine the possible role of genes belonging to the upregulated DEGs list identified in our *in-silico* analyses, we performed growth screens using *S. cerevisiae* deletion mutants that were grown in coculture with *L. thermotolerans*. More than 200 DEGs needed to be screened, which presented a major challenge. Large volume fermentations were impractical for the screening, and instead we made use of a reasonably high throughput method. However, screening cocultures in high throughput is challenging. Only a few studies in such attempts have been published, both detailing challenges in generating reproducible data (Conacher et al., 2024; Pourcelot et al., 2023). None attempted the large number of screened samples required in our case. Therefore, we assessed the feasibility of microtiter plate-based cocultures to score interaction-relevant growth defects in small scale coculture fermentations. Phenotypic data on *S. cerevisiae* genes in small scale monoculture fermentations exists, such as the arrayed deletion mutant libraries of *S. cerevisiae* laboratory strains BY4741 and BY4742 used in this study, which provide a reference point when assessing the role of these genes in more complex ecosystems.

**Figure 4:**
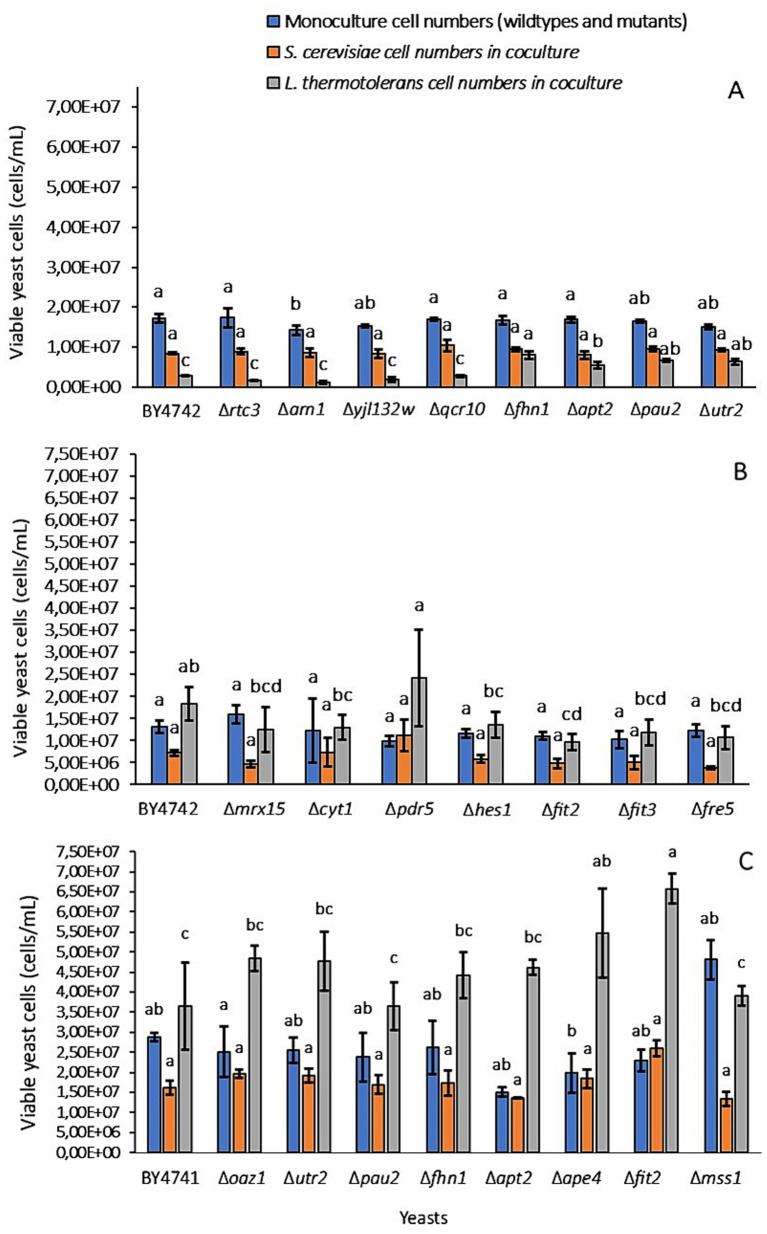
Phenotypic profiles for screens that were performed by coculturing wildtype *S. cerevisiae* BY4741 and BY4742, or mutants of these strains, with *L. thermotolerans* for 12 h in ISA-SGM. Each profile consists of a *S. cerevisiae* monoculture (blue bars) and a coculture pairing (orange and grey bars) of *S. cerevisiae* and *L. thermotolerans*. Viable yeast cell numbers (cells/mL) were measured for monocultures (blue bars) of the wildtypes BY4741 or BY4742 and the mutant strains derived from these yeasts. For the cocultures, cell numbers of *S. cerevisiae* wildtypes or mutants (orange bars) and the numbers of *L. thermotolerans* (grey bars) cells in these pairings were measured. The *S. cerevisiae* deletion mutants that were screened in cocultures included **(A)** Δ*rtc3* to Δ*utr2*, **(B)** Δ*mrx15* to Δ*fre5,* and **(C)** Δ*oaz1* to Δ*mss1*.

**Figure 5:**
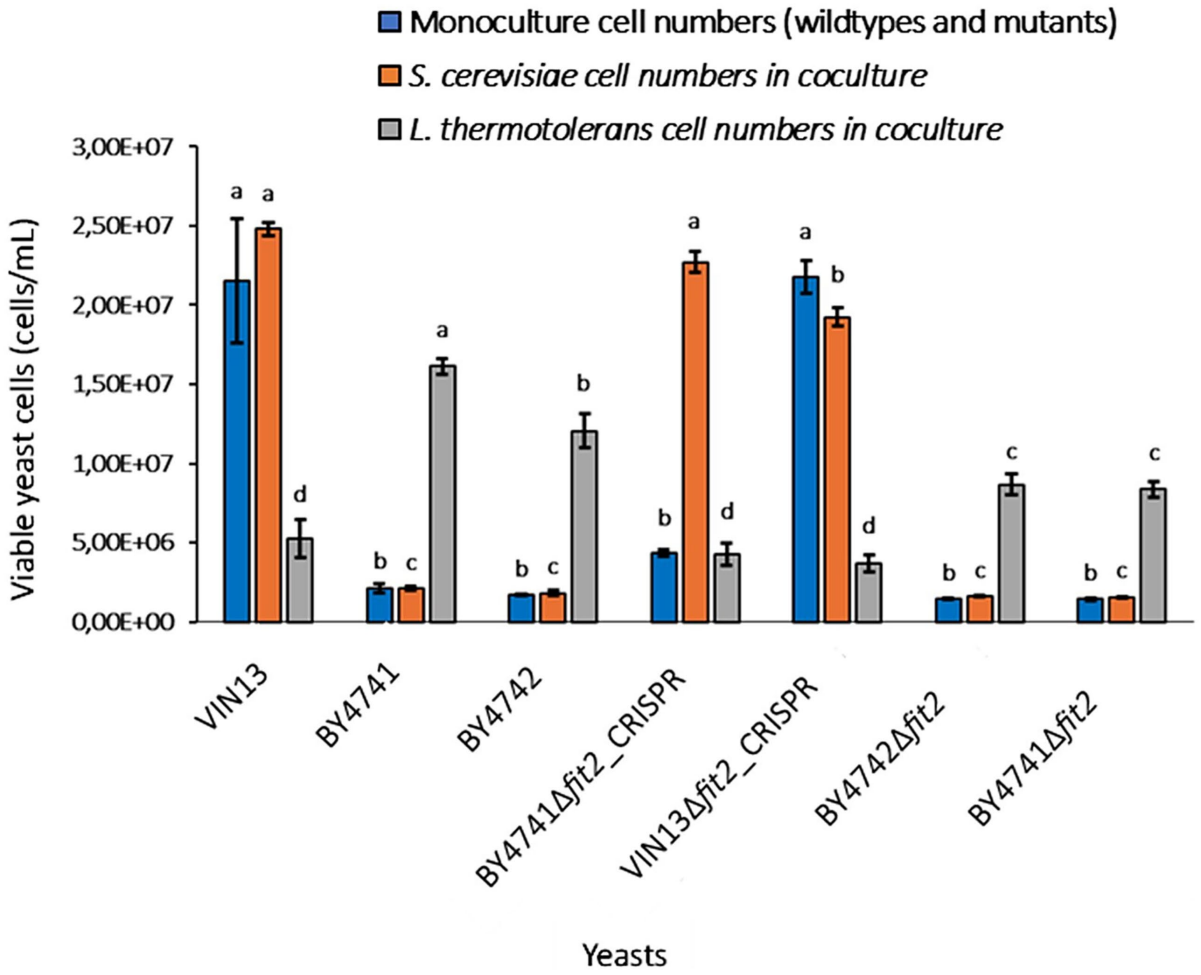
Phenotypic profiles for screens that were performed by coculturing wildtype and mutant *S. cerevisiae* strains with *L. thermotolerans* for 12 h in ISA-SGM. Each profile consists of an *S. cerevisiae* monoculture (blue bars) and a coculture pairing (orange and grey bars) of *S. cerevisiae* and *L. thermotolerans*. Viable yeast cell numbers (cells/mL) were measured for *S. cerevisiae* monocultures (blue bars); including wildtypes of VIN13 (wine strain), BY4741 and BY4742, *FIT2* deletion mutants of these yeasts from deletion libraries (e.g., BY4741Δ*fit2* and BY4742Δ*fit2*), and *FIT2* knock-out mutants that were created in this study (e.g., BY4741Δ*fit2*_CRISPR and VIN13Δ*fit2*_CRISPR). For the cocultures, cell numbers of *S. cerevisiae* wildtypes or mutants (orange bars) and the numbers of *L. thermotolerans* (grey bars) cells in these pairings were measured.

However, while the outcome of phenotypic screening tests in monocultures are generally relatively easy to score quantitatively, the addition of competition between yeasts and fluctuating environmental parameters makes phenotypes more difficult to evaluate in a multispecies context (Conacher et al., 2020; Kemsawasd et al., 2015; Pourcelot et al., 2023). In addition, as the available deletion libraries represent laboratory strains, the evolutionary relevance of observed screening phenotypes or phenotypic impacts of gene deletion on wine ecosystem interactions remain uncertain. Nonetheless, we hypothesized that at least some interaction relevant genes would be revealed through interaction phenotypes related to gene deletion. Each screening set included a WT *S. cerevisiae* strain of the mutant library that was used or the wine strain VIN13, which served as controls for comparisons (Figures 4, 5; Supplementary Figure S14). Growth phenotypes of yeasts in coculture pairings were scored by measuring the viable cell number differences (*p*-value < 0.05) between mono- and cocultures.

The 12 h coculture time point was used to score the phenotypes, since this time point has seen growth of both species, while providing sufficient time for potential interaction-relevant phenotypes to impact growth performance. Later time points tended to diverge between replicates, making scoring less accurate, while earlier time points tended to reflect monoculture growth. Monoculture controls of each deletion mutant were included in each screen. This was done to ensure that any cell number differences between *S. cerevisiae* mutants and WT strains were not related to inherent growth rate differences. Yeasts were cultured in monocultures (blue bars), as well as mixed cultures of *S. cerevisiae* (orange bars) and *L. thermotolerans* (grey bars) for deletion mutants that were screened including (A) Δ*rtc3* to Δ*utr2*, (B) Δ*mrx15* to Δ*fre5* and (C) Δ*oaz1* to Δ*mss1*. These pairings were compared to the coculture phenotype profile of the WT strains (e.g., BY4741 or BY4742). In these screenings, targets for further investigation were defined as those belonging to phenotypic profiles that had significant differences in “*L. thermotolerans* cocultures” viable cell numbers but did not show differences in their “monoculture” cell numbers.

All available viable deletion mutants in libraries for 203 genes from up- and downregulated DEG lists were screened in coculture with *L. thermotolerans*, however, not all screens were performed in triplicate. These data appear in the Supplementary material and were not considered for statistical evaluation, as these *L. thermotolerans*/mutant pairings were used for rapid indications of potential interaction relevance and require additional validation (Supplementary Figures S11, S12). It should be noted that data for Figures 4, 5 were derived from measurements of three biological repeats. The first screen that was performed consisted of four coculture pairings of deletion mutants with *L. thermotolerans* (Figure 4). Monoculture cell numbers of these mutants were similar (1.50- to 1.67 × 10^7^ cells/mL). However, the viable *L. thermotolerans* cell numbers (e.g., > 5 × 10^6^ cells/mL) in cocultures involving mutants were lower compared to monoculture (1.72 × 10^7^ cells/mL) and coculture *L. thermotolerans* cell numbers (2.80 × 10^6^ cells/mL) with the BY4742 control.

There were no significant differences (*p*-value > 0.05) regarding cell numbers of *S. cerevisiae* deletion mutants, BY4742 control and *L. thermotolerans* for the screening involving mutants of Δ*pms1* to Δ*stl1* (Supplementary Figure S14A), while further screenings that were performed also revealed no differences regarding *L. thermotolerans* coculture cell numbers (Supplementary Figures S14B,C). However, deletion mutants Δ*yol131w* and Δ*orm2* (1.58 × 10^7^ cells/mL and 8.55 × 10^6^ cells/mL respectively) showed significantly lower (*p*-value < 0.05) monoculture cell numbers than BY4742 (2.01 × 10^7^ cells/mL). Δ*fit2* deletion mutant/*L. thermotolerans* cocultures had significantly lower *L. thermotolerans* cell numbers (9.70 × 10^6^ cells/mL) compared to the numbers of this yeast (1.82 × 10^7^ cells/mL) when paired with BY4742 WT (Figure 4B).

Deletion mutants of Δ*rtc3* and Δ*utr2* genes were rescreened (Figures 4A; Supplementary Figure S14) in BY4742 deletion library screenings as growth trends were inconsistent. Mutants from the BY4741 deletion library collection, e.g., Δ*oaz1* to Δ*mss1* and Δ*met* representatives, were also used in our screenings (Figures 4C; Supplementary Figure S14D). Deletion of the *MET* genes, specifically genes represented by deletion mutants Δ*met3/10/14* and *17*, had no significant impact (*p*-value > 0.05) on viable cell numbers of *S. cerevisiae* mutant monocultures or *L. thermotolerans* in cocultures with these yeasts compared to the WT BY4741 reference mono- and cocultures (Supplementary Figure S14D).

On the contrary, Δ*ape4* and Δ*fit2* (5.47 and 6.57 × 10^7^ cells/mL respectively) showed significant differences (*p*-value < 0.05) regarding cell numbers for *L. thermotolerans* cocultures compared to BY4741 WT cocultures (3.65 × 10^7^ cells/mL) (Figure 4C). To validate the impact of certain genes on *S. cerevisiae*/*L. thermotolerans* coculture growth dynamics observed in the screenings, *FIT2* was selected for CRISPR-Cas9 mediated gene-knockouts in *S. cerevisiae* VIN13 and BY4741 WT. This selection was based on consistent differences in screening observations for Δ*fit2* mutants, as these coculture pairings significantly affected (*p*-value < 0.05) *L. thermotolerans* cell numbers in cocultures compared to growth with either of the WT references (e.g., BY4741 and BY4742). In addition, as *FIT2* is a cell wall protein, many of which have been implicated in yeast interactions, it made this gene an attractive candidate given that the cell wall is at the interface of cell–cell interactions.

### CRISPR-Cas9 knock-out of FIT2 in *S. cerevisiae* VIN13 and BY4741 strains

To confirm the effect of *FIT2* deletion in *S. cerevisiae* on population dynamics of cocultures involving this yeast and *L. thermotolerans*, this gene was knocked out in a known diploid *S. cerevisiae* wine strain (e.g., VIN13) (Borneman et al., 2011) and the haploid BY4741 WT using CRISPR-Cas9 gene editing and strains were screened as before. Successful deletion of *FIT2* was confirmed in representative transformants of these yeasts by visualizing fragments obtained from PCR amplification of mutants and a WT control (VIN13) as well as sequencing of purified amplification products (Supplementary Figure S13; Supplementary Text S1). Thereafter, coculture screenings were performed with confirmed transformants and WT controls, e.g., VIN13, BY4741 and BY4742 (Figure 5).

The WT reference for each *S. cerevisiae* yeast strain tested in coculture with *L. thermotolerans* (e.g., VIN13, BY4741 and BY4742) were included for comparisons of phenotypic profiles to Δ*fit2* mutants. Experimental conditions and data points were the same as described in Figure 4, e.g., an inoculation density of 2:0.5 (2 × 10^6^ cells/mL:0.5 × 10^6^ cells/mL) was used for mono- and mixed cultures of *S. cerevisiae*: L*. thermotolerans* that were grown in 220 μL ISA-SGM in microplates at 600 rpm between 22 and 25 °C, and were scored at 12 h sampling point. No significant differences (*p*-value > 0.05) in viable cell numbers were observed for monoculture and *L. thermotolerans* coculture populations of the Δ*fit2* deletion mutant for VIN13 (VIN13Δ*fit2*_CRISPR) compared to VIN13 WT.

However, *S. cerevisiae* coculture cell numbers were significantly lower (*p*-value < 0.05) in the mutant (e.g., 1.92 × 10^7^ cells/mL) versus the WT (e.g., 2.47 × 10^7^ cells/mL). WTs of BY4741 and BY4742 showed no difference (*p*-value > 0.05) regarding their monoculture and *S. cerevisiae* coculture cell numbers. In addition, *L. thermotolerans* cell numbers in coculture with BY4741 were significantly higher (e.g., 1.92 × 10^7^ cells/mL; *p*-value < 0.05) than when this yeast was cocultured with BY4742 (e.g., 1.20 × 10^7^ cells/mL). No differences in cell numbers for mono- or cocultures between Δ*fit2* mutant strains belonging to either of the deletion libraries (e.g., BY4741Δ*fit2* and BY4742Δ*fit2*). In contrast, *L. thermotolerans* coculture cell numbers were significantly lower (3.70 × 10^6^ cells/mL; *p*-value < 0.05) in the *FIT2* knock-out mutant of BY4741 (e.g., BY4741Δ*fit2*_CRISPR) that was created compared to BY4741Δ*fit2* and BY4742Δ*fit2* library mutants (8.36- and 8.67 × 10^6^ cells/mL respectively). In addition, the BY4741Δ*fit2*_CRISPR mutant also had significantly higher (*p*-value < 0.05) *S. cerevisiae* coculture cell numbers (2.27 × 10^7^ cells/mL) than the deletion library representatives (1.56- and 1.61 × 10^6^ cells/mL for BY4741Δ*fit2* and BY4742Δ*fit2* respectively) and VIN13Δ*fit2*_CRISPR mutant (1.92 × 10^7^ cells/mL).

## Discussion

This work aimed at identifying transcriptional signals that are specifically linked to interspecies interactions between *S. cerevisiae* and *L. thermotolerans* during coculture fermentations. Therefore, a pooled analysis approach was taken in which an *in-silico* pipeline was used to re-analyze datasets from previous studies. Datasets were re-evaluated and compared under standardized settings with this pipeline, which reduced the impact of confounding variables from the distinct conditions, strains and reference states that were used in each separate study (Figure 2). The results from the analysis aligned with and reinforced earlier findings from these studies (Conacher et al., 2022; Luyt et al., 2024; Shekhawat et al., 2019). This suggests our findings could indicate the existence of species-specific transcriptional profiles and possibly genes governing ecological interactions in *S. cerevisiae* (Supplementary Table S2). In addition, it demonstrates how the approach followed in this work for analysis can be useful in identifying enriched processes and their associated DEGs for further exploration. This sequence of analysis is also followed in recent comparative transcriptional studies that assessed *S. cerevisiae* pathways responding to the presence of non-*Saccharomyces* yeast species (Fu et al., 2024; Mejias-Ortiz et al., 2025). Interestingly, exposure of *S. cerevisiae* to extracellular vesicles (EVs) of other wine yeast species, or coculturing of this yeast with *L. thermotolerans* in sour beer fermentations, revealed similar pathway responses in *S. cerevisiae.* This included up-regulation of genes related to ribosome biogenesis, while enriched downregulated biological processes were related to inorganic cation transmembrane transport.

Biological processes involving ion transport, e.g., cation transport, were found to be highly enriched and upregulated in *S. cerevisiae* (Supplementary Table S2). In addition, the datasets that were reassessed in our work involved cultures of cells in direct contact with one another, which resulted in expression of identified DEGs indicating a link between physical cell–cell contact and differential gene expression (Conacher et al., 2022; Luyt et al., 2024; Shekhawat et al., 2019). Indeed, more evidence to support this link can be seen by the fact that many of these DEGs were cell wall genes, e.g., members of *PAU* gene family, are believed to be involved in interspecies yeast interactions (Brou et al., 2018; Luyt et al., 2024; Rivero et al., 2015; Tronchoni et al., 2017). *FIT2* was among the most upregulated genes in coculture conditions and encodes a mannoprotein in the yeast cell wall involved in uptake and retention of siderophore-iron in the cell wall (Philpott et al., 2002; Protchenko et al., 2001). Expression of *FIT2* is known to be induced under iron-deprivation, but this gene was also suggested to be activated by glycolysis and has appeared in other studies involving *S. cerevisiae/L. thermotolerans* mixed fermentations (Luyt et al., 2024; Shekhawat et al., 2019; Wu et al., 2004). SGM that was used in studies re-analyzed in our pooled analysis contains limited amounts of available iron for yeasts (Supplementary Table S1). *FIT* gene expression under iron deprivation was proposed to assist cells to store usable iron for periods of extreme iron deprivation, as subtle modulation of iron uptake could significantly impact yeast survival under these conditions (Protchenko et al., 2001).

It is plausible that *FIT2* carries out a similar function in coculture interactions occurring between *S. cerevisiae* and *L. thermotolerans* during fermentation. Indeed, other studies have suggested that similar strategies are employed by certain yeast species, such as *Metschnikowia pulcherrima* and *Rhodotorula glutinis*, to provide these yeasts with a competitive advantage over other fungi in their environments (Calvente et al., 1999; Sipiczki, 2006; Wang et al., 2020). This is achieved by the production of either pulcherriminic acid or rhodotorulic acid, respectively, by each species which act as either ferric iron chelators or siderophores to scavenge available iron from the environment thereby inhibiting growth of competitors in ecosystems. *S. cerevisiae* inhibits *L. thermotolerans* growth in mixed fermentations (Conacher et al., 2022; Conacher et al., 2020; Luyt et al., 2024; Luyt et al., 2021), while *FIT2* expression was found to be either highly up- or downregulated in cocultures of these yeasts in different studies (Luyt et al., 2024; Shekhawat et al., 2019).

Each study made use of different wine strains of *S. cerevisiae*, suggesting that regulation of this gene may be strain-dependent. This could explain why we see a negative impact on *S. cerevisiae* coculture growth in the VIN13Δ*fit2* mutant compared to the VIN13 WT, while simultaneously observing stimulated *S. cerevisiae* growth in BY4741Δ*fit2*_CRISPR cocultures compared to BY4741 and BY4742 WT controls, mutants and VIN13Δ*fit2* (Figure 5). To determine if the phenotype observed for *FIT2* mutant interactions is strain-specific and if this gene is involved in an antagonistic iron sequestration coculture interaction the screening should be repeated and scored using the same *S. cerevisiae* strains that have been modified to overexpress *FIT2*.

It is worth mentioning that, as *FIT2* is a known component of the cell wall, deletion could have unknown impacts on the expression of other cell wall gene families. For example, *FLO* and *PAU* gene expression were linked to coaggregation and environmental stress, e.g., cell–cell contact, experienced by yeasts in *S. cerevisiae*/*L. thermotolerans* cocultures and could be partly responsible for the observed phenotypes (Luyt et al., 2024; Rossouw et al., 2018). With this in mind, it is important to note that the cell wall composition differs between industrial and laboratory strains of *S. cerevisiae*. Laboratory strains, such as BY4741, lack the *FLO8* gene which acts as a transcriptional activator needed to induce *FLO* gene expression (Liu et al., 1996). However, wine strains (e.g., VIN13) possess this gene and expression of individual *FLO* genes in commercial strains was found to produce strikingly different flocculation phenotypes (Govender et al., 2010). Indeed, it was demonstrated that expression of various *FLO* genes in VIN13 caused increased cell surface hydrophobicity compared to the WT that naturally possesses a low level of surface hydrophobicity.

This trait is important as it influences co-flocculation levels between yeast cells, which was shown to influence population dynamics in cocultures of *S. cerevisiae* and *L. thermotolerans* (Rossouw et al., 2018). Therefore, while no significant differences (*p*-value > 0.05) were observed for *L. thermotolerans* cell numbers in cocultures with VIN13Δ*fit2* mutant compared to VIN13 WT screens, *S. cerevisiae* coculture numbers between these phenotypic profiles were impacted. This stresses the importance of monitoring both yeast populations in the screening strategy and further supports that *FIT2* expression (see Text S1 for confirmation of deletion) is impacting coculture interactions between these yeasts (Figure 5). It also emphasizes that strain-related differences likely impact phenotypic profiles between cocultures of *L. thermotolerans* and either *S. cerevisiae* VIN13 or BY4741 wild type and mutant strains. Additionally, another explanation for the divergence in phenotypic profiles observed for our recreated BY4741Δ*fit2*_CRISPR knockout strain compared to BY4741Δ*fit2*/BY4742Δ*fit2* library representatives could be related to unforeseen secondary mutations present in the mutant library strains. This could also explain variations in phenotypic profiles observed for different mutant strains, e.g., BY4741Δ*fit2* and BY4742Δ*fit2*, in coculture with *L. thermotolerans* (Figures 4B,C) (Gros et al., 2009; Protchenko et al., 2001). Clearly, *S. cerevisiae*/*L. thermotolerans* coculture interactions involving *FIT2* are complex, and a combination of many factors may contribute to resultant phenotypes.

Indeed, our findings for *FIT2* highlighted that even though defined conditions were used for coculture screenings, it remains difficult to score “true” interaction phenotypes accurately. Phenotypic data generated for other mutant cocultures did not provide indications of links between identified DEGs and their impact on *S. cerevisiae*/*L. thermotolerans* coculture interactions. Many optimizations, e.g., scaling, media selection, inoculation density and sampling times, were carried out during the development of the method before we reached the final conditions that were implemented (Figures 4, 5; Supplementary Figures S5–S10). These optimizations improved consistency, reproducibility and standard error rate used to score phenotypes, while also assisting in determining a reasonable time for allowing interaction between yeasts to occur before sampling. Other small volume screening strategies, which included strains that appear in our work, e.g., *S. cerevisiae* VIN13 mCherry and *L. thermotolerans* IWBT Y1240 BFP (Conacher et al., 2024; Pourcelot et al., 2023), demonstrate how common the challenges experienced in our work are in terms of reproducibility of results when studying yeast cocultures.

Arguably the greatest influence regarding reproducibility in these studies, and possibly our work, could be inoculation density inaccuracies when working with small volume fermentations (Conacher et al., 2024). The impact that slight differences in inoculation ratios can have on observed coculture phenotypes is demonstrated in recent work involving the construction of syntrophic yeast pairings (Park et al., 2024). To construct interspecies syntrophic communities, *S. cerevisiae* auxotrophs were paired with auxotrophs of *Yarrowia lipolytica*. This revealed *S. cerevisiae: Y. lipolytica* auxotrophic pairings (such as SCΔ*trp2*-YLΔ*trp4*) inoculated at dosages of 1:1, 1:5 and 1:10 were able to grow and showed similar growth trends, while dosage ratios of 10:1 and 5:1 did not grow. Additionally, recent work demonstrated the importance of inoculation ratios in modulating wine acidification and fermentation kinetics during *S. cerevisiae*/*L. thermotolerans* coculture fermentations (Vicente et al., 2025b). These factors need to be considered in mixed fermentations, as certain co-inoculation ratios were linked to increased media acidification. Consequently, increased acidity levels observed for some coculture combinations were suggested to have caused a decline in *S. cerevisiae* implantation percentages in cocultures, while a co-inoculation ratio of 10:1 (*L. thermotolerans*: *S. cerevisiae*) was found to yield optimum lactic acid levels, while maintaining proper fermentative kinetics. This highlights the impact that adjustments in yeast ratios can have in coculture systems.

Inoculation densities and timepoints tested in our study during optimizations, including 1:1, 2:1 and 2:0.5 (Supplementary Figures S9, S10), noticeably impacted *L. thermotolerans* biomass in phenotypic profiles obtained for cocultures of this yeast with deletion mutants. It is therefore possible that results obtained for some of the mutant screenings described here can be explained by small differences in inoculation density (Figures 4; Supplementary Figure S14). Future studies should consider incorporating additional tools, such as machine learning, to allow for more rapid and reliable differentiation and quantification of *L. thermotolerans*/*S. cerevisiae* mutant cocultures. This addition may assist in addressing issues faced throughout our screening approach. An example of such an approach was the recent coupling of multicolor flow cytometry to a machine learning classifier, e.g., a Gaussian Mixture Model combined with Random Forest classifier (Midani and David, 2023). This allowed researchers to easily determine absolute abundance of different *Bacteroides* species in cocultures. Alternatively, imaging flow cytometry could be considered, which was used to differentiate and simultaneously count fluorescently stained yeast and bacteria, e.g., *S. cerevisiae* and *Lactiplantibacillus plantarum*, in mixed cultures prepared at various cell ratios during sour beer fermentations (Williamson et al., 2023).

In summary, the data highlight the relevance of investigating molecular systems for their role in ecosystem and interaction relevant phenotypes. Indeed, many molecular genetic functions have likely evolved in response to ecosystem specific selection pressures. This type of analysis can place genetic functions within their appropriate evolutionary context which has been rather neglected due to the single species bias of most microbiological analysis. However, the data also highlight the challenges involved in such approaches, including the lack of appropriate high-throughput phenotyping tools for such studies. Furthermore, studying binary coculture phenotypes and their molecular components under synthetic conditions may result in different type of biases. Contrarily to single species studies focusing on molecular mechanisms, binary interaction studies indeed may yield very different phenotypes depending on specific strains and their evolutionary history.

## Data Availability

The studies are listed in our article which contain the data repositories and accession numbers. However, this information is as follows in chronological order for each study: Data repositories: National Centre for Biotechnology Information (NCBI) Gene Expression Omnibus (GEO) and Sequence Read Archive (SRA) databasesAccession numbers: GSM3073202-GSM3073211; PRJNA783452; PRJNA902701.

## Footnotes

1
https://bioinformatics.psb.ugent.be/webtools/Venn

2 Addgene plasmid #90962; http://n2t.net/addgene:90962; RRID: Addgene_90,962
