# Correction: Investigating the transcriptional fingerprints of cocultured *Saccharomyces cerevisiae* and *Lachancea thermotolerans* in a model wine environment

**DOI:** 10.3389/fmicb.2026.1802169

**Published:** 2026-02-23

**Authors:** Justin Joseph Asmus, Rene Kathleen Naidoo-Blassoples, Roberto Pérez-Torrado, Florian F. Bauer

**Affiliations:** 1 Department of Viticulture and Oenology, South African Grape and Wine Research Institute, Stellenbosch University, Stellenbosch, South Africa; 2 Instituto de Agroquímica y Tecnología de los Alimentos, IATA-CSIC, Paterna, Spain

**Keywords:** coculture, *FIT2*, interactions, pooled analysis *Lachancea thermotolerans*, *Saccharomyces cerevisiae*

The funder MCIN/AEI/10.13039/501100011033, PID2022-142748OB-I00 to Roberto Pérez-Torrado was erroneously omitted.

The corrected Funding statement is below:

The author(s) declared that financial support was received for this work and/or its publication. The following funders supported this research: The National Research Foundation of South Africa through Grant no. 83471 (SA Research Chair in Integrated Wine Science) to FB. Funding was also received from the Spanish National Research Council (CSIC) for the “i-COOP 2021” program for scientific cooperation and development under Modality A with the call reference COOPA20491. This publication is part of the R + D + i project PID2022-142748OB-I00, financed by MCIN/AEI/10.13039/501100011033 to RP-T. The Accreditation as Center of Excellence Severo Ochoa CEX2021-001189-S funded by MCIU/AEI/10.13039/501100011033 is also fully acknowledged.

The original version of this article has been updated.

