# Supplementary material for: Investigating the transcriptional fingerprints of cocultured Saccharomyces cerevisiae and Lachancea thermotolerans in a model wine environment
Source: Front Microbiol. 2026 Jan 2;16:1720597. doi: 10.3389/fmicb.2025.1720597 (PMC12807978; doi:10.3389/fmicb.2025.1720597)
Supplement: Supplementary file 1 [file Table_1.DOCX]

**Table S1** SGM and ISA-SGM base media components and their final concentrations prior to yeast inoculations.

| **Amino acids** | **Concentration (g/L)** |
| --- | --- |
| Tyrosine | 1.400 |
| Tryptophan | 13.700 |
| Isoleucine | 2.500 |
| Aspartic acid | 3.400 |
| Glutamic acid | 9.200 |
| Arginine | 28.600 |
| Leucine | 3.700 |
| Threonine | 5.800 |
| Glycine | 1.400 |
| Glutamine | 38.600 |
| Alanine | 11.100 |
| Valine | 3.400 |
| Methionine | 2.400 |
| Phenylalanine | 2.900 |
| Serine | 6.000 |
| Histidine | 2.500 |
| Lysine | 1.300 |
| Cysteine | 1.000 |
| Proline | 46.800 |
| **Viatmins** | **Concentration (mg/L)** |
| Myo-inositol | 100.000 |
| Calcium pantothenate | 1.000 |
| Thiamine hydrochloride | 0.500 |
| Nicotinic acid | 2.000 |
| Para-aminobenzoic acid (PABA) | 0.200 |
| Riboflavin | 0.200 |
| Folic acid | 0.200 |
| Pyrodoxine hydrochloride | 2.000 |
| Biotin | 0.125 |
| **Trace elements** | **Concentration (µg/L)** |
| Co(NO_3_)_2_·6H_2_O | 30.000 |
| CuCl_2_ | 15.000 |
| H_3_BO_3_ | 5.000 |
| FeCl_2_ | 30.000 |
| MnCl_2_·4H_2_O | 200.000 |
| KIO_3_ | 10.000 |
| NaMoO_4_.2H_2_O | 25.000 |
| ZnCl_2_ | 135.000 |

**Table S2** Gene set enrichment analysis obtained using ShinyGO depicting the top 10 enriched pathways relating to overlapping up- and downregulated genes in *S. cerevisiae* cultured in mixed direct cell-cell contact fermentations with *L. thermotolerans* that were generated by this study. Gene ontology (GO) terms relating to biological processes were selected for the analysis. The false discovery rate (FDR) corrected enrichment score, number of genes (nGenes) present in our gene list that was input and the number of genes in the enriched pathway, the fold-enrichment of the pathway, as well as the enriched pathway and gene names corresponding to our input list are shown in the table below.

| **Upregulated gene list** | | | | | |
| --- | --- | --- | --- | --- | --- |
| **Enrichment FDR** | **nGenes** | **Pathway  Genes** | **Fold  Enrichment** | **Pathway** | **Genes** |
| 2.78E-04 | 4 | 11 | 32.43 | GO:0015677 copper ion import | *FRE5, CTR3, FRE7, CTR1* |
| 4.16E-07 | 10 | 65 | 13.72 | GO:0000041 transition metal ion transport | *ARN1, ZRT3, ZRT2, FRE7, ENB1, FRE5, FIT2, FIT3, CTR3, CTR1* |
| 2.78E-04 | 6 | 41 | 13.05 | GO:0006826 iron ion transport | *ARN1, FRE7, ENB1, FRE5, FIT2, FIT3* |
| 2.67E-05 | 10 | 102 | 8.74 | GO:0030001 metal ion transport | *ARN1, ZRT3, ZRT2, FRE7, ENB1, FRE5, FIT2, FIT3, CTR3, CTR1* |
| 1.94E-04 | 9 | 104 | 7.72 | GO:0055076 transition metal ion homeostasis | *ARN1, CTR3, FRE7, ENB1, FRE5, CTR1, FIT2, FIT3, ZRT3* |
| 4.16E-07 | 17 | 251 | 6.04 | GO:0006812 cation transport | *ARN1, ZRT3, ZRT2, FRE7, ENB1, FRE5, FIT2, FIT3, STL1, RIP1, QCR10, CTR3, COX5A, PHM7, CYT1, CTR1, FAA4* |
| 1.83E-04 | 11 | 163 | 6.02 | GO:0098662 inorganic cation transmembrane transport | *ZRT3, ZRT2, STL1, RIP1, ARN1, QCR10, CTR3, COX5A, ENB1, CYT1, CTR1* |
| 2.45E-04 | 12 | 209 | 5.12 | GO:0098655 cation transmembrane transport | *ZRT3, ZRT2, STL1, RIP1, ARN1, QCR10, CTR3, COX5A, PHM7, ENB1, CYT1, CTR1* |
| 4.16E-07 | 20 | 357 | 5.00 | GO:0006811 ion transport | *ARN1, ZRT3, AQY2, ZRT2, FRE7, ENB1, FRE5, FIT2, FIT3, STL1, RIP1, QCR10, YHK8, CTR3, COX5A, PHM7, CYT1, CTR1, FAA4* |
| 1.64E-04 | 14 | 268 | 4.66 | GO:0034220 ion transmembrane transport | *ZRT3, AQY2, ZRT2, STL1, RIP1, ARN1, QCR10, CTR3, COX5A, PHM7, ENB1, CYT1, CTR1* |

**Table S2 (continued)**

| **Downregulated gene list** | | | | | |
| --- | --- | --- | --- | --- | --- |
| **Enrichment FDR** | **nGenes** | **Pathway  Genes** | **Fold  Enrichment** | **Pathway** | **Genes** |
| 2.67E-07 | 10 | 39 | 13.65 | GO:0009086 methionine biosynthetic proc. | *MET6, MET10, ADE3, ARO9, BAT1, MET28, MET3, MET5, MET14, MET17* |
| 3.42E-07 | 10 | 41 | 12.98 | GO:0000097 sulfur amino acid biosynthetic proc. | *MET10, MET5, MET6, ADE3, ARO9, BAT1, MET28, MET3, MET14, MET17* |
| 3.71E-07 | 11 | 54 | 10.84 | GO:0000096 sulfur amino acid metabolic proc. | *MET6, MET10, MET5, MET32, ADE3, ARO9, BAT1, MET28, MET3, MET14, MET17* |
| 4.11E-07 | 11 | 55 | 10.65 | GO:0009067 aspartate family amino acid biosynthetic proc. | *ARO9, ASN1, MET6, MET10, ADE3, BAT1, MET28, MET3, MET5, MET14, MET17* |
| 5.62E-09 | 18 | 120 | 7.98 | GO:1901607 alpha-amino acid biosynthetic proc. | *LEU2, ARO9, BAT1, GDH1, GLN1, ASN1, ILV6, SER3, MET6, MET10, ADE3, MET28, MET3, MET5, MET14, MET17, CAR2, CIT2* |
| 1.73E-08 | 18 | 135 | 7.10 | GO:0008652 cellular amino acid biosynthetic proc. | *LEU2, ARO9, BAT1, GDH1, GLN1, ASN1, ILV6, SER3, MET6, MET10, ADE3, MET28, MET3, MET5, MET14, MET17, CAR2, CIT2* |
| 1.73E-08 | 21 | 197 | 5.67 | GO:0016053 organic acid biosynthetic proc. | *ILV6, LEU2, ADE3, ARO9, BAT1, GDH1, EEB1, GLN1, ASN1, MET10, MET5, SER3, MET6, MET28, MET3, MET14, MET17, CAR2, DLD3, ALD5, CIT2* |
| 1.73E-08 | 21 | 197 | 5.67 | GO:0046394 carboxylic acid biosynthetic proc. | *LEU2, ADE3, ARO9, BAT1, GDH1, EEB1, GLN1, ASN1, ILV6, SER3, MET6, MET10, MET28, MET3, MET5, MET14, MET17, CAR2, DLD3, ALD5, CIT2* |
| 2.58E-07 | 19 | 186 | 5.44 | GO:1901605 alpha-amino acid metabolic proc. | *LEU2, MET6, ARO9, BAT1, CAR2, YGP1, GDH1, GLN1, ASN1, ILV6, SER3, MET10, ADE3, MET28, MET3, MET5, MET14, MET17, CIT2* |
| 2.74E-07 | 29 | 445 | 3.47 | GO:0006082 organic acid metabolic proc. | *ILV6, LEU2, GLK1, EMI2, DLD3, MET6, HXK1, PYC1, ADE3, ARO9, BAT1, PHO90, MAE1, CAR2, YGP1, GDH1, EEB1, GLN1, ASN1, MET10, MET5, MET32, CIT2, SER3, MET28, MET3, MET14, MET17, ALD5* |

| **Upregulated gene list** | | | | | |
| --- | --- | --- | --- | --- | --- |
| **Enrichment FDR** | **nGenes** | **Pathway Genes** | **Fold Enrichment** | **Pathway** | **Genes** |
| 4.54E-02 | 6 | 22 | 6.20 | Ergosterol biosynthetic process | *C5DEH6, C5DF95, C5DFP3, C5DIY6, C5DM14, C5E3M7* |
| 4.54E-02 | 6 | 22 | 6.20 | Phytosteroid biosynthetic process | *C5DEH6, C5DF95, C5DFP3, C5DIY6, C5DM14, C5E3M7* |
| 4.54E-02 | 6 | 22 | 6.20 | Cellular alcohol biosynthetic process | *C5DEH6, C5DF95, C5DFP3, C5DIY6, C5DM14, C5E3M7* |
| 4.54E-02 | 6 | 23 | 5.93 | Ergosterol metabolic process | *C5DEH6, C5DF95, C5DFP3, C5DIY6, C5DM14, C5E3M7* |
| 4.54E-02 | 6 | 23 | 5.93 | Phytosteroid metabolic process | *C5DEH6, C5DF95, C5DFP3, C5DIY6, C5DM14, C5E3M7* |
| 4.74E-02 | 6 | 24 | 5.68 | Fatty acid biosynthetic process | *C5DDV5, C5DFB0, C5DGZ5, C5DIE6, C5DM66, C5E297* |
| 4.54E-02 | 9 | 49 | 4.18 | Monocarboxylic acid biosynthetic process | *C5DDV5, C5DFB0, C5DGZ5, C5DHQ3, C5DIE6, C5DL36, C5DM66, C5E297, C5E361* |
| 9.46E-06 | 44 | 405 | 2.47 | Oxidation-reduction process | *C5DC53, C5DDE2, C5DDI2, C5DDV5, C5DE71, C5DE89, C5DEG6, C5DEH6, C5DES6, C5DF95, C5DFB0, C5DFP3, C5DFW3, C5DGD0, C5DGM4, C5DGP5, C5DID0, C5DIE6, C5DIZ8, C5DJ02, C5DJ14, C5DL36, C5DLN2, C5DLZ5, C5DM14, C5DM16, C5DM18, C5DM41, C5DM66, C5DMB3, C5DMB5, C5DMM6, C5DMU1, C5DMW4, C5DNA1, C5DND7, C5DNH6, C5DNQ4, C5DNS1, C5E1Z3, C5E1Z5, C5E240, C5E297, C5E396* |

**Table S3** Gene set enrichment analysis obtained using ShinyGO depicting the top 10 enriched pathways relating to overlapping up- and downregulated genes in L. thermotolerans cultured in mixed direct cell-cell contact fermentations with S. cerevisiae generated by this study. Gene ontology (GO) terms relating to biological processes were selected for the analysis. The false discovery rate (FDR) corrected enrichment score, number of genes (nGenes) present in our gene list that was input and the number of genes in the enriched pathway, the fold-enrichment of the pathway, as well as the enriched pathway and gene names corresponding to our input list are shown in the table columns below.

**Table S3 (continued)**

| **Downregulated gene list** | | | | | |
| --- | --- | --- | --- | --- | --- |
| **Enrichment FDR** | **nGenes** | **Pathway Genes** | **Fold Enrichment** | **Pathway** | **Genes** |
| 1.95E-05 | 6 | 8 | 21.22 | De novo imp biosynthetic process | *C5DBK4, C5DD99, C5DI18, C5DIY2, C5DJD4, C5DLT0* |
| 4.14E-05 | 6 | 10 | 16.98 | IMP biosynthetic process | *C5DBK4, C5DD99, C5DI18, C5DIY2, C5DJD4, C5DLT0* |
| 4.14E-05 | 6 | 10 | 16.98 | IMP metabolic process | *C5DBK4, C5DD99, C5DI18, C5DIY2, C5DJD4, C5DLT0* |
| 1.95E-05 | 8 | 17 | 13.32 | Purine nucleoside monophosphate biosynthetic process | *C5DBK4, C5DD99, C5DFK7, C5DH49, C5DI18, C5DIY2, C5DJD4, C5DLT0* |
| 2.33E-05 | 8 | 19 | 11.91 | Purine ribonucleoside monophosphate metabolic process | *C5DBK4, C5DD99, C5DFK7, C5DH49, C5DI18, C5DIY2, C5DJD4, C5DLT0* |
| 2.33E-05 | 10 | 33 | 8.57 | Ribonucleoside monophosphate biosynthetic process | *C5DBK4, C5DCU6, C5DD99, C5DFK7, C5DH49, C5DI18, C5DIY2, C5DJD4, C5DKM3, C5DLT0* |


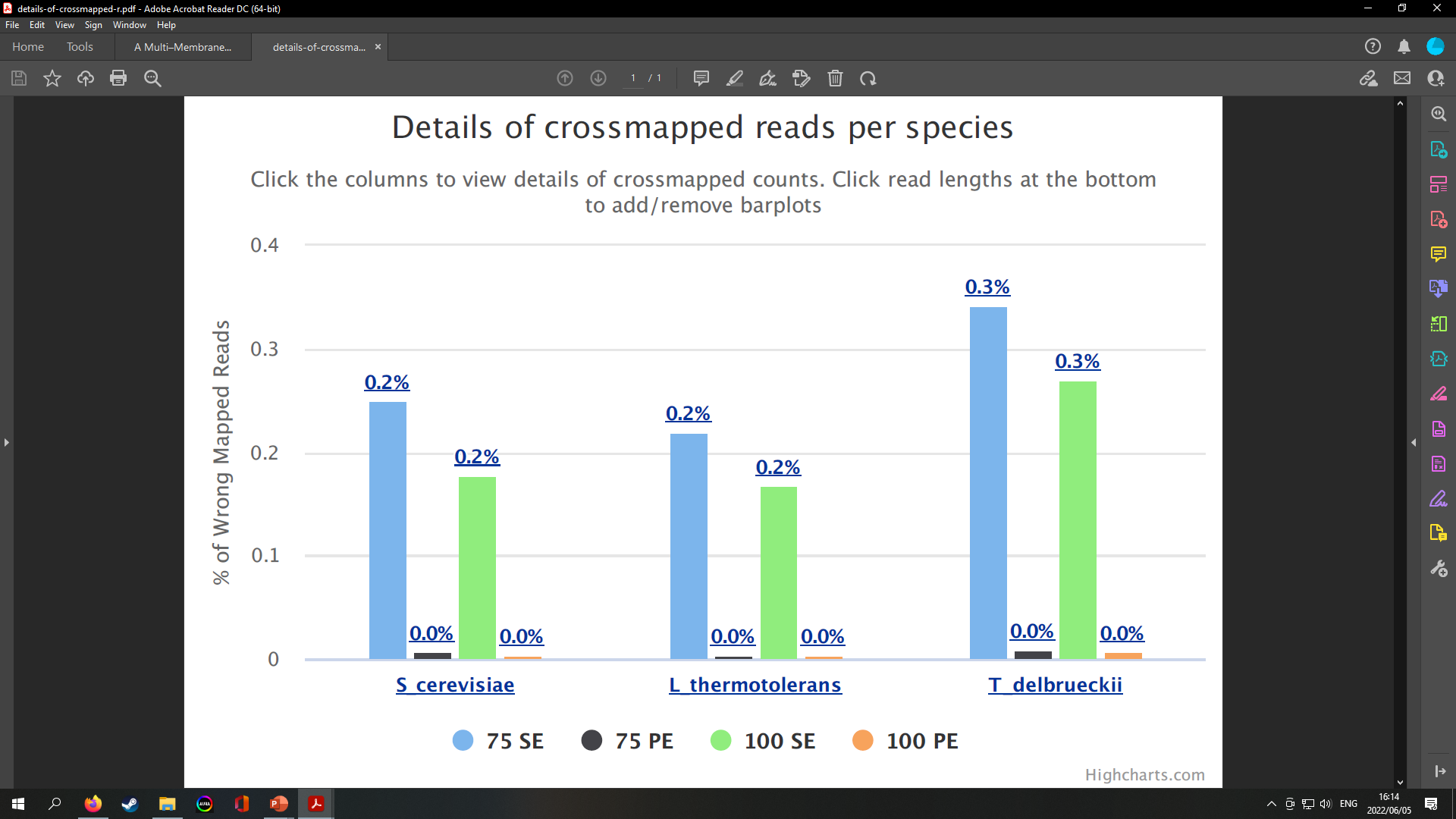


**Figure S1** Plot depicting the percentage of simulated transcriptome reads that were found to map non-specifically to reference genomes of either Saccharomyces cerevisiae or Lachancea thermotolerans. In this analysis, default parameters were used to generate single end (SE) or paired end (PE) pseudoreads of either 75- or 100-basepairs (bp) that gave sufficient transcriptome coverage for read mapping. Both read types (SE and PE), and different lengths thereof (75 and 100 bp), resulted in less than 1% incorrect unique and multimapped reads to non-corresponding genomes of the yeasts that were tested.

**Figure S2** Venn diagrams representing comparisons performed for differentially expressed genes (DEGs) of L. thermotolerans that were obtained from differential gene expression analyses described in detail in the methods section. Gene lists that were compared were the same as mentioned for the S. cerevisiae comparisons that were conducted including; (A) upregulated genes from datasets of cocultures of L. thermotolerans and S. cerevisiae in a continuous fermentation environment under aerobic (Lt_AR) and anaerobic (Lt_AN) conditions (Shekhawat et al., 2019), cocultures from a membrane bioreactor study where cells were cultured under direct cell-cell contact for 2-h (LtMD2h) and 24-h (LtMD24h) as well as culturing restricted to metabolic exchange (physically separated cells e.g. MI_UP and MI_DOWN) (Luyt et al., 2024), and batch fermentations where yeasts were cultured under aerated conditions for 7-h (C_Lt) (Conacher et al., 2022). (B) Gene lists comparing the downregulated genes for analyses that were conducted with the same annotations mentioned in A. The final comparisons that were performed served to filter genes that overlapped with those also present under physical cell separation (e.g. MI_UP versus MD_UP and MI_DOWN versus MD_DOWN), to acquire genes relating to physical contact between cells which reflected screening conditions.

**B**


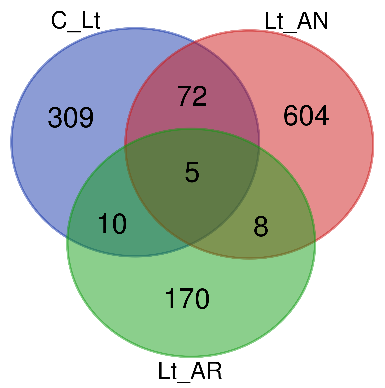

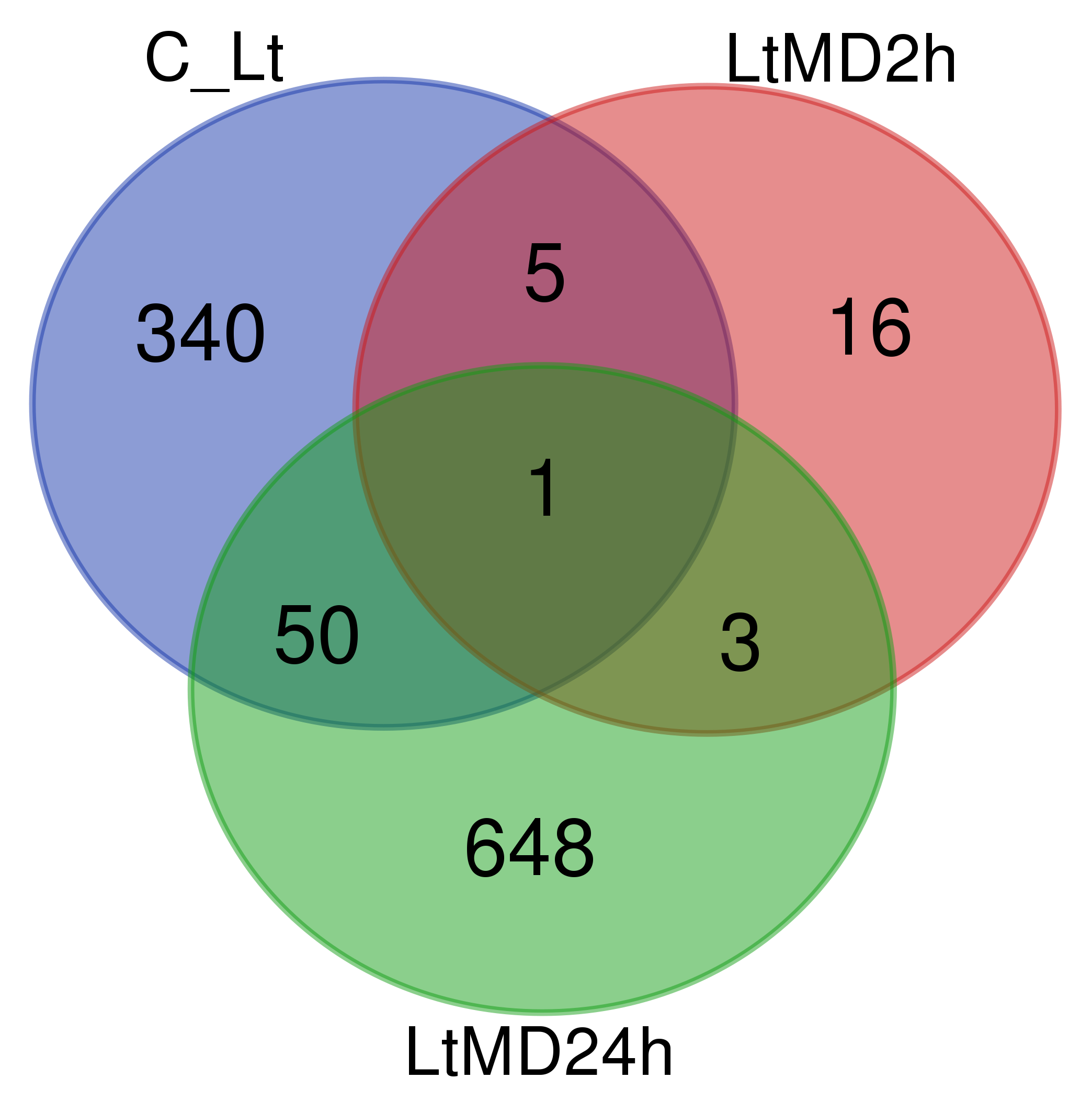

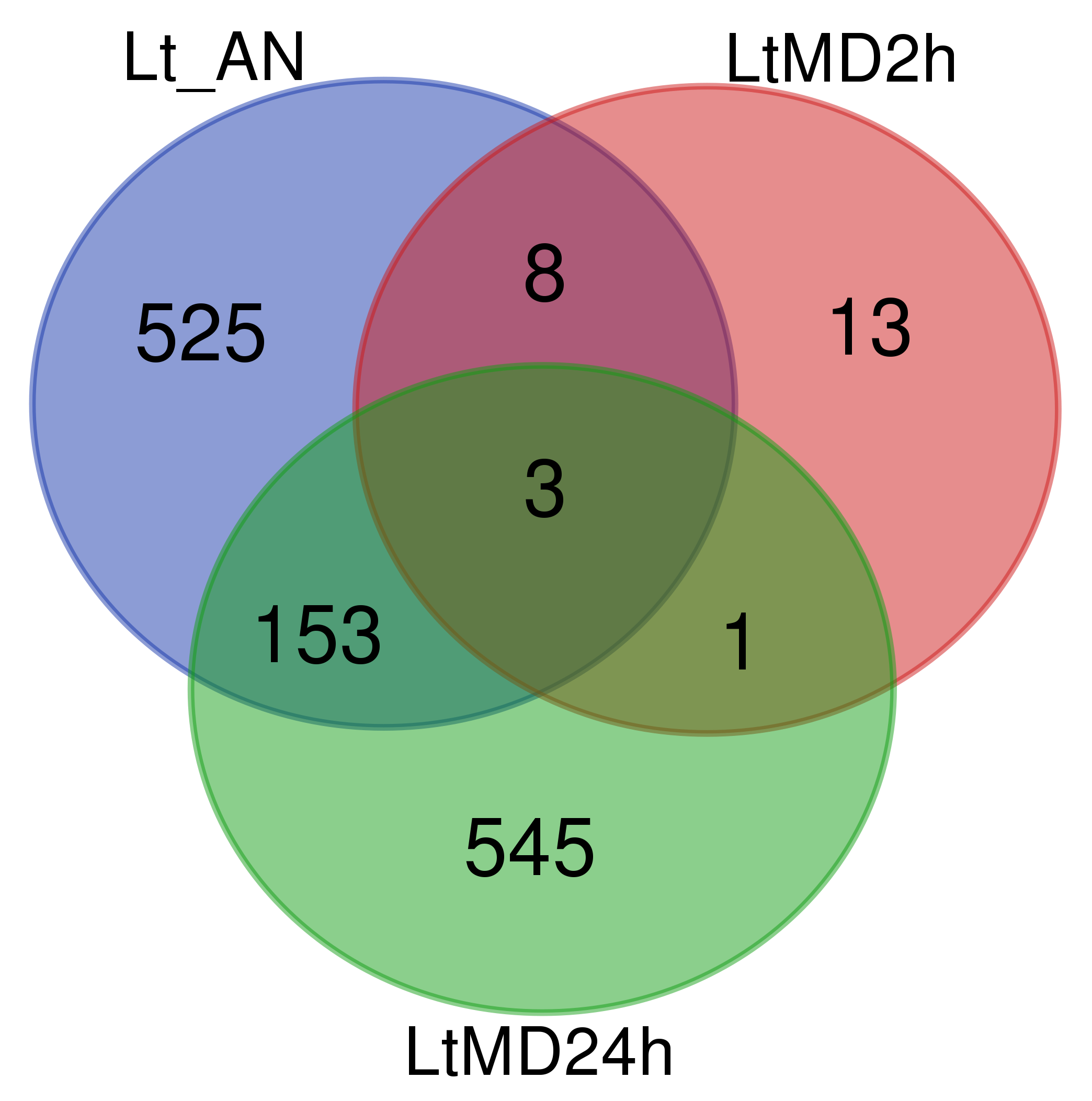

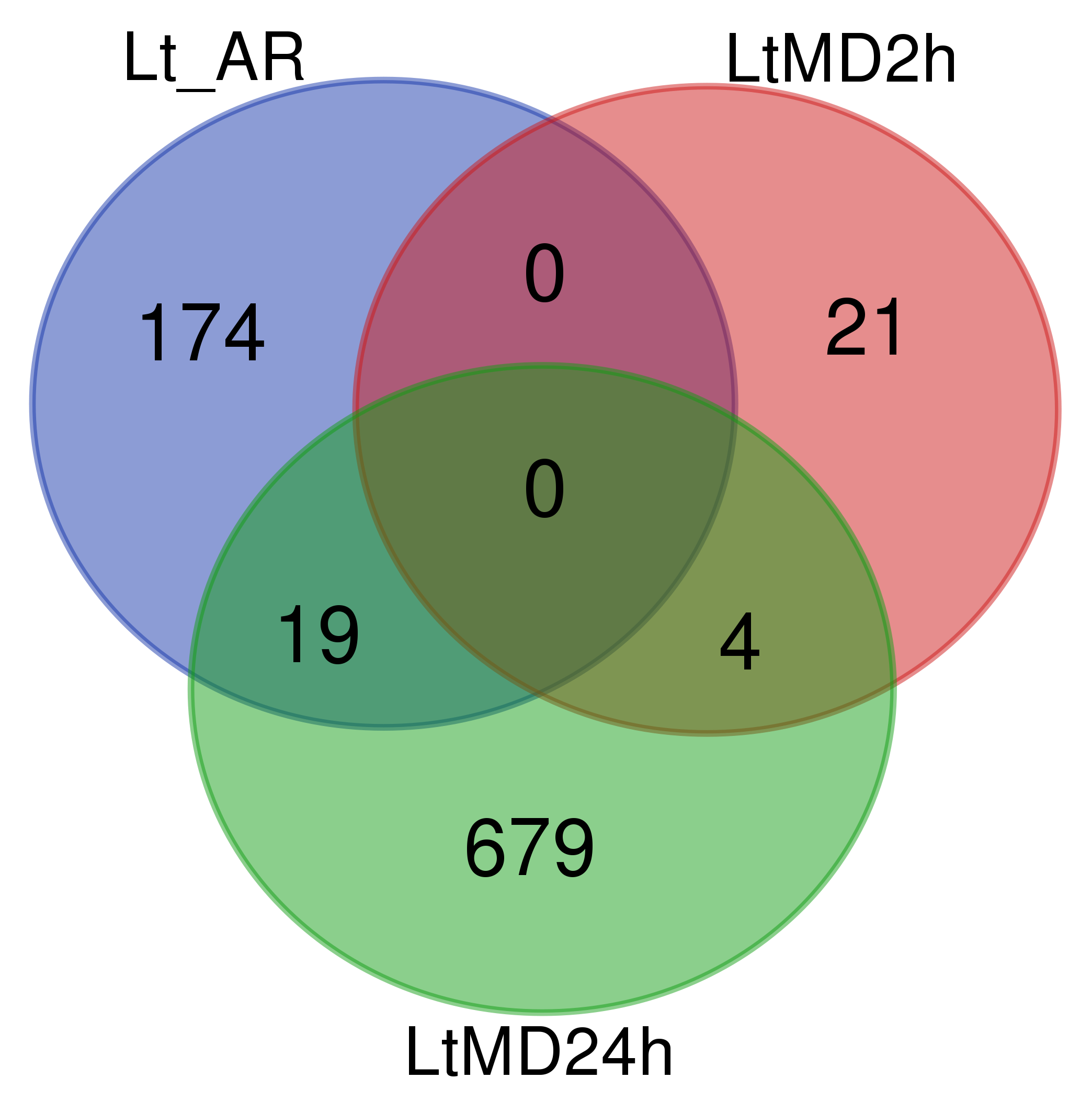


**A**


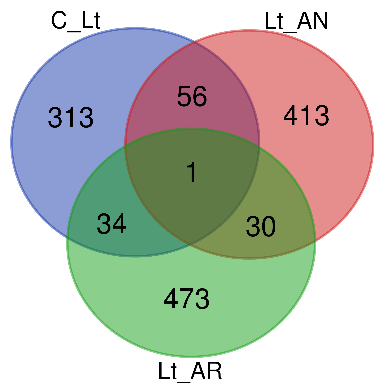

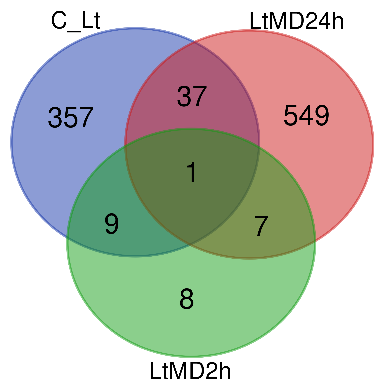

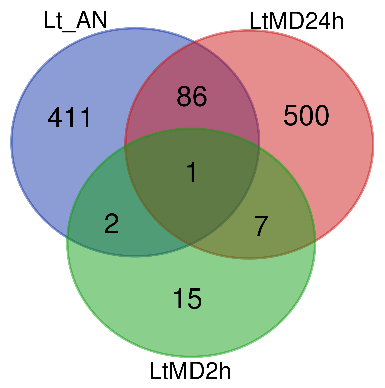

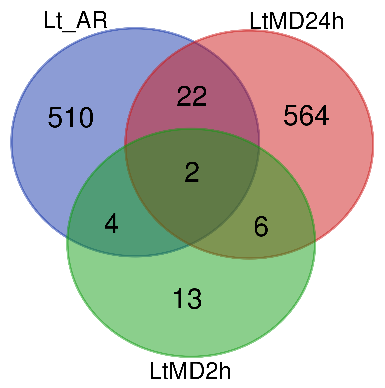

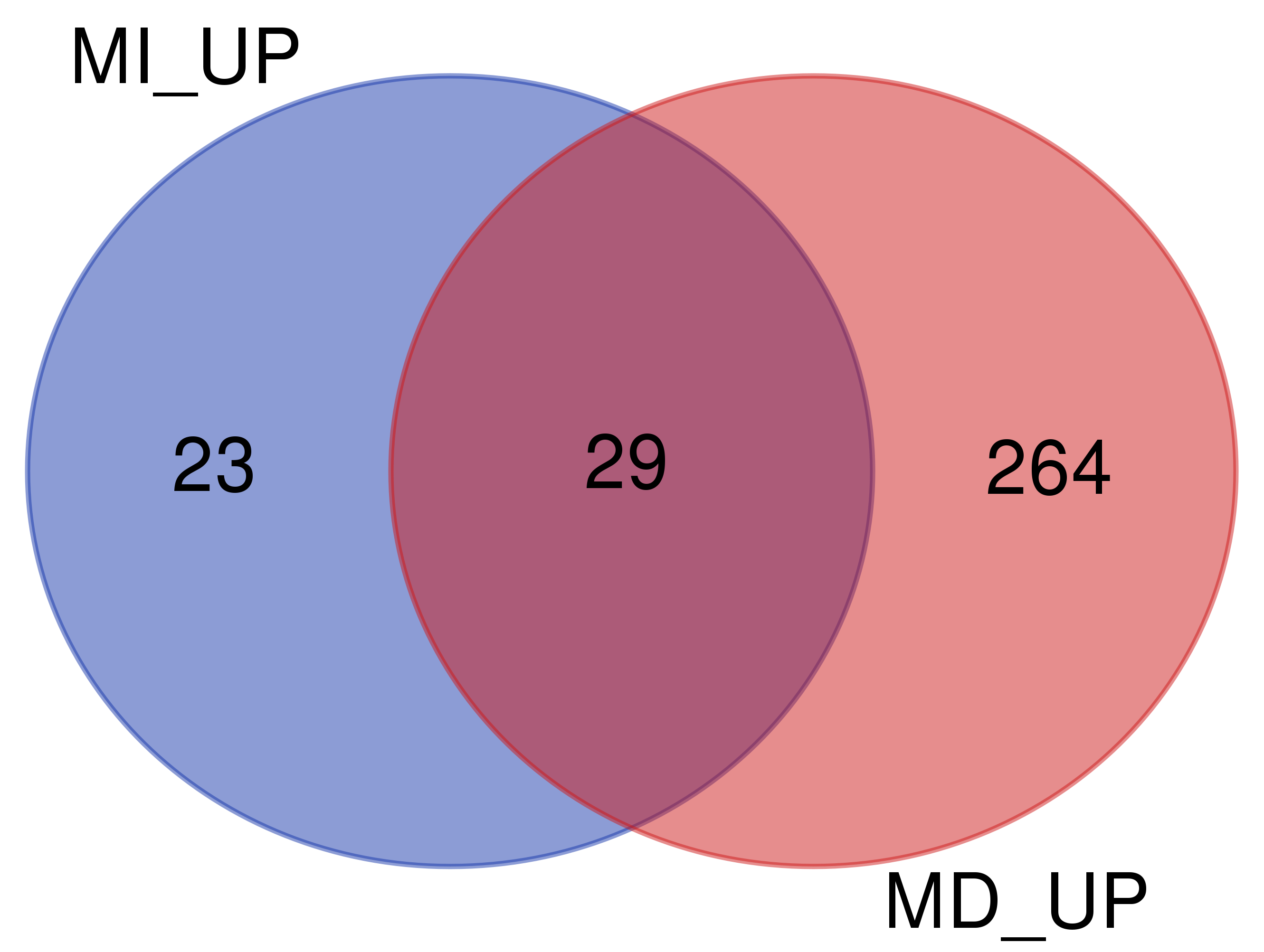

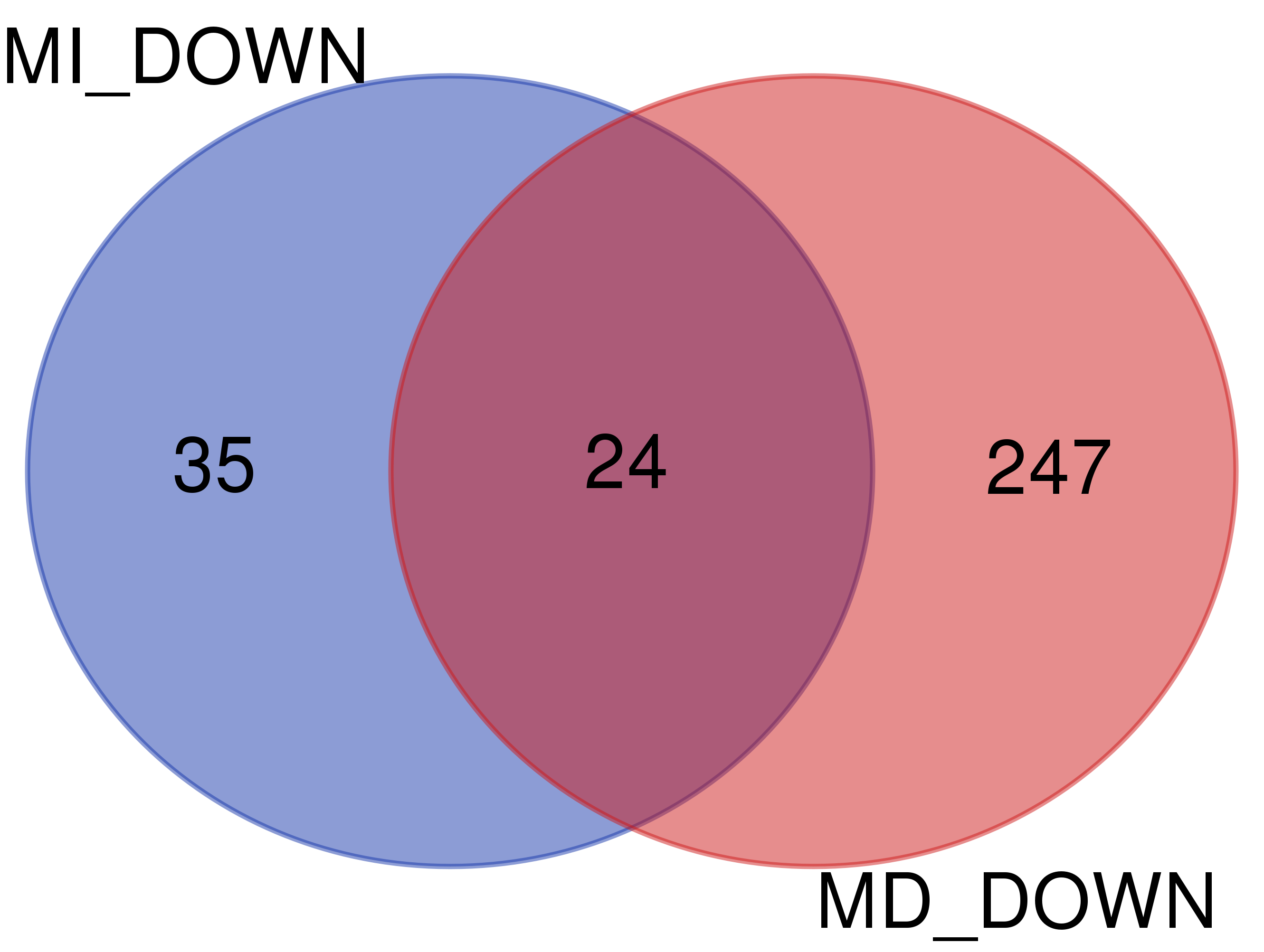


**Figure S3A** Hierarchical dendrograms summarizing clusters of significant GO terms for (A) upregulated and (B) downregulated gene lists that were analysed. The larger circles (blue) at the end of branches correspond to terms with more significant FDR values (p-value < 0.05). Terms are grouped according to the number of shared genes that they possess.

**Figure S3B** Hierarchical dendrograms summarizing clusters of significant GO terms for (A) upregulated and (B) downregulated gene lists that were analysed for L. thermotolerans. The larger circles (blue) at the end of branches correspond to terms with more significant FDR values (p-value < 0.05). Terms are grouped according to the number of shared genes that they possess.


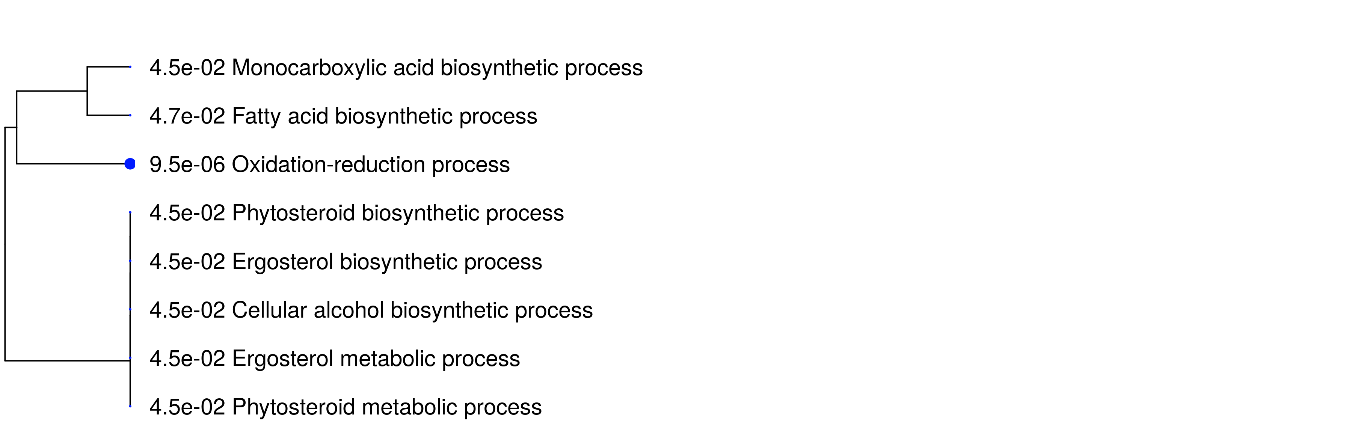

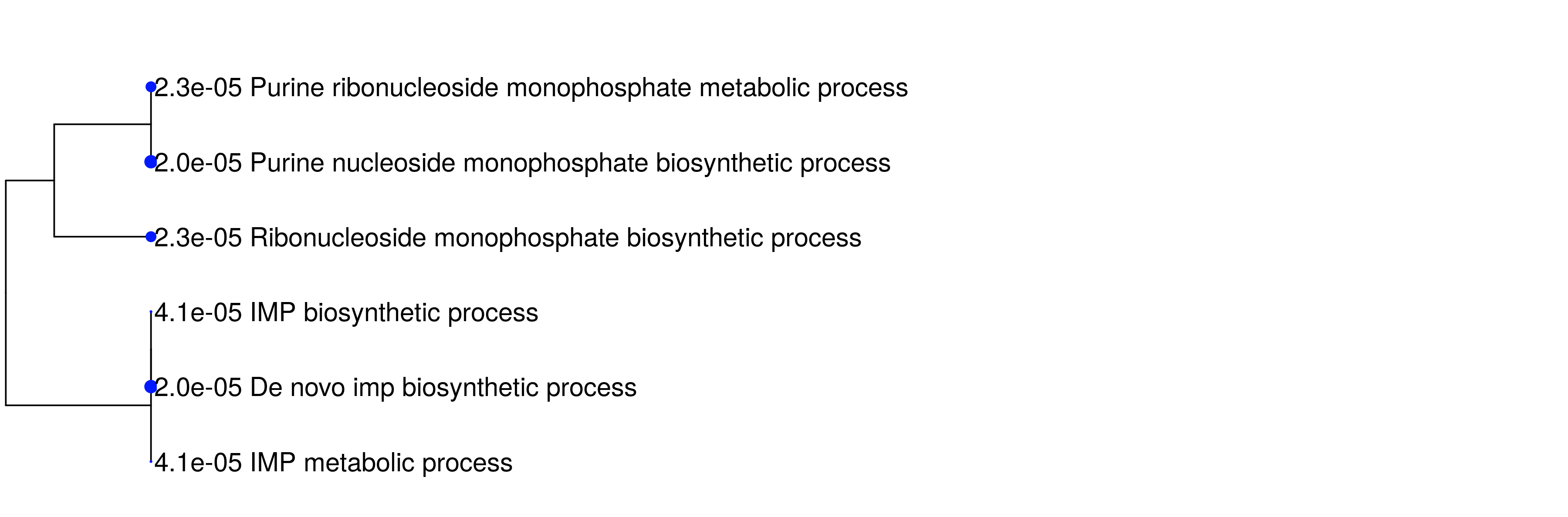


**A**

**B**


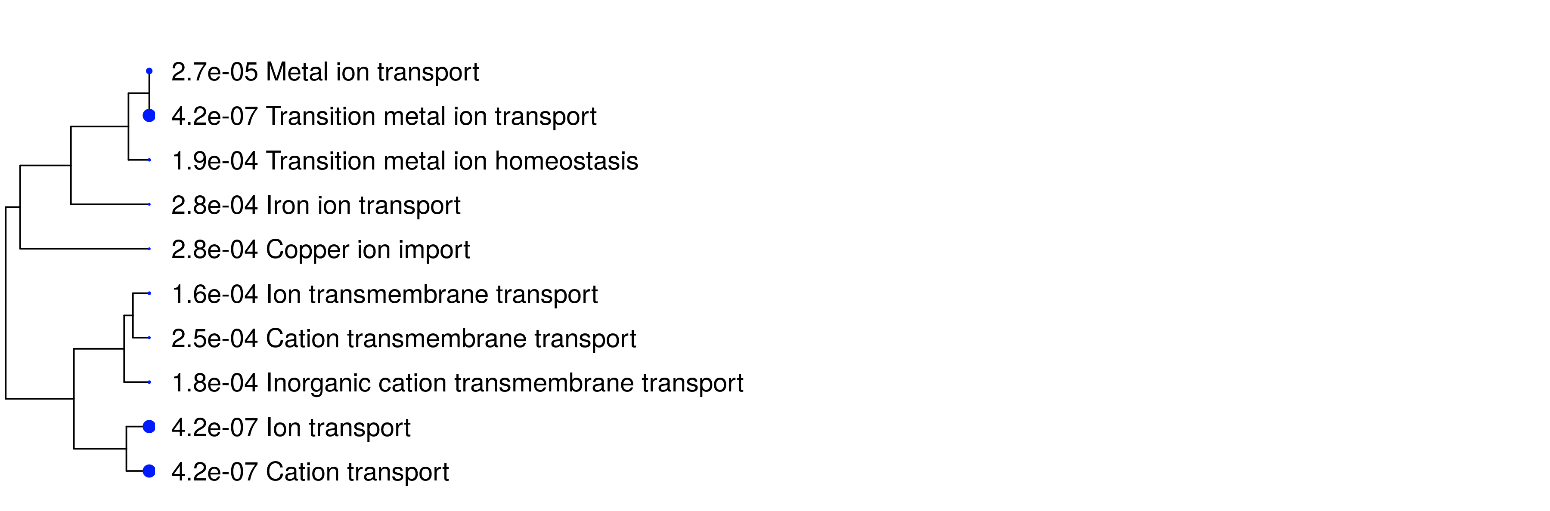

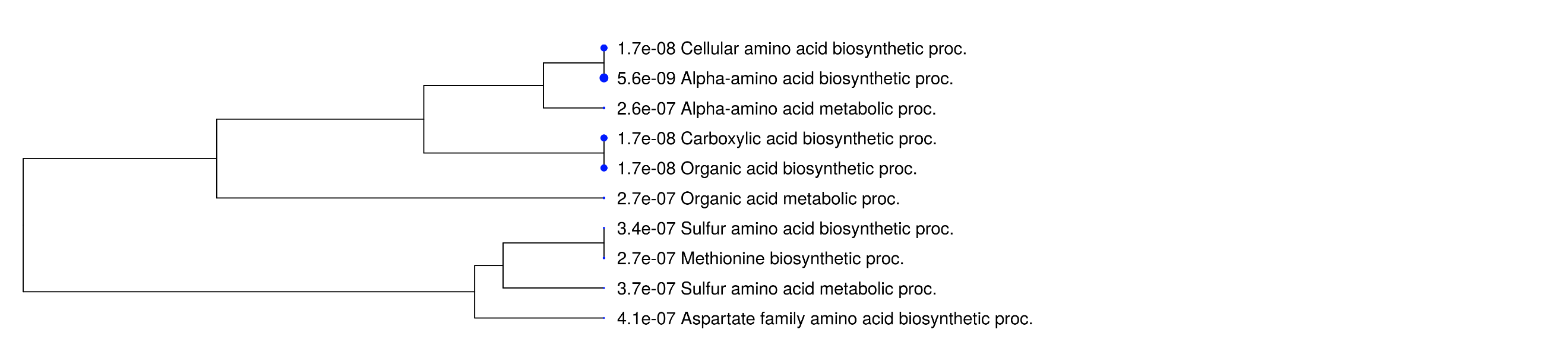


**A**

**B**

**Figure S5** Representatives of S. cerevisiae BY4742 deletion mutant library strains (missing gene name indicated) that were pre-cultured in 5 mL YPD test tubes at 40 rpm on rotary shaker wheel for roughly 20-h at room temperature (ca. 25°C). Thereafter, yeast cultures were harvested (5000 x g; 20 °C; 5 min), inoculated (1 × 10^6^ viable cells/mL) into ISA-SGM and further cultured in a 96-well microplate using a VICTOR Nivo multimode plate reader for 100 kinetic cycles, where bi-directional measurements were taken every 15 min after agitation of cells (600 rpm, double-orbital shaking, 1 min 30 s duration).

**Figure S4** Comparison of plate counts (CFU/mL) on WL agar and viable yeast cell (cells/mL) data (cells stained with propidium iodide (PI) 1µM) for monocultures of the fluorescently labelled Lachancea thermotolerans IWBT Y1240 and Saccharomyces cerevisiae BY4742 (wildtype) cultured in 5 mL ISA-SGM test tubes at 40 rpm on rotary shaker wheel for 24-h at 30°C.

**Figure S7A** Deletion mutant strain representatives and wildtype of S. cerevisiae BY4742 in coculture with L. thermotolerans after pre-culturing as described in Figure S5. Yeast cultures were then harvested, inoculated and cultured for 24-h in a 96-well microplate using a VICTOR Nivo multimode plate reader under the same conditions mentioned in Figure S5, where BY4742 wildtype and mutants were inoculated in a 1:1 ratio with L. thermotolerans in cocultures (e.g. 1 × 10^6^ viable cells/mL:1 × 10^6^ viable cells/mL). The left-hand vertical y-axis indicates the viable yeast cells (cells/mL) after PI staining (1µM) for monocultures of BY4742 wildtype and mutants (blue bars), as well as cell numbers of pairings of these yeasts (S. cerevisiae co-culture, orange bars) and L. thermotolerans (L. thermotolerans co-culture, grey bars) in mixed fermentations seen on the horizontal axis. The secondary vertical axis on the right-hand side of the plot indicates the endpoint absorbance values (λ = 600 nm, dashed line) for the monocultures of each tested strain after 24-h. The bars and absorbance values represent the mean of biological triplicates accompanied by standard error in each case.

**Figure S6** Deletion mutant strain representatives of S. cerevisiae BY4742 seen in Figure S5 pre-cultured in 5 mL ISA-SGM under the same conditions. Thereafter, yeast cultures were harvested, inoculated and further cultured in ISA-SGM as mentioned in Figure S5.

**Figure S8** Differences in viable yeast cell numbers after 24-h growth between separate batches of ISA-SGM, e.g. Monocultures_New_ISA-SGM and Monocultures_Old_ISA-SGM, that were prepared according to modifications to SGM detailed in the methods section of our study. Deletion mutant representative strains and wildtype of S. cerevisiae BY4742 were cultured according to the same procedures mentioned for the monocultures in Figure S7B. The mean of three biological replicates with standard error is indicated for each yeast culture.

**Figure S7B** Repeated culturing of the deletion mutant strain representatives and wildtype of S. cerevisiae BY4742 in coculture with L. thermotolerans as described in Figure S7A, however, here the yeast cultures were cultivated on a digital microplate shaker (Thermo Fisher Scientific, Waltham, MA, USA) in ISA-SGM for 24-h at room temperature (ca. 22 -25°C) under constant agitation at 600 rpm. The endpoint absorbance (λ = 600 nm, dashed line) for each tested strain is again indicated on the secondary vertical axis on the right-hand side.

**B**


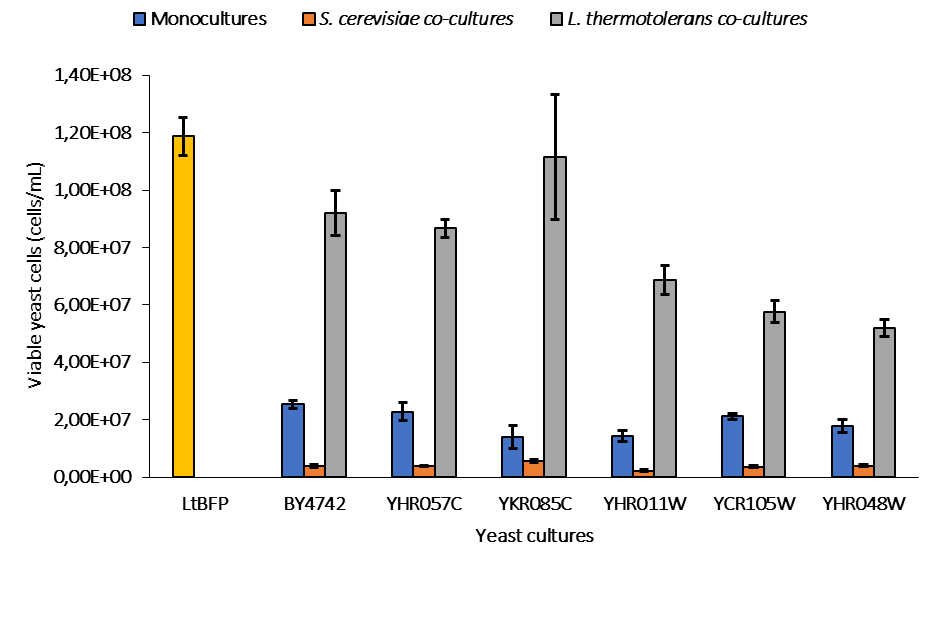

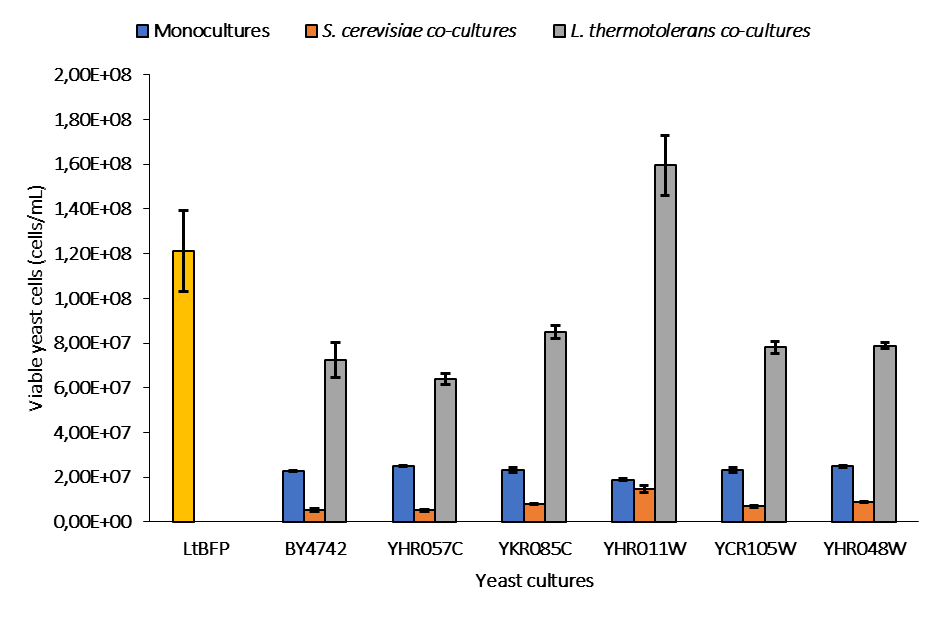


**A**

**B**

**Figure S9** Differences in viable yeast cell numbers after 24-h growth in ISA-SGM between deletion mutant strain representatives and wildtype of S. cerevisiae BY4742 in coculture with L. thermotolerans under the same conditions described in Figure S7B. (A) Monocultures and L. thermotolerans control (“LtBFP” yellow bar) were inoculated into ISA-SGM at an initial cell density of 1 × 10^6^ viable cells/mL, while the cocultures consisted of a 1:1 ratio (e.g. 1 × 10^6^ viable cells/mL:1 × 10^6^ viable cells/mL) S. cerevisiae:L. thermotolerans. (B) S. cerevisiae monocultures were inoculated into ISA-SGM at an initial cell density of 2 × 10^6^ viable cells/mL and L. thermotolerans control (“LtBFP” yellow bar) was inoculated at 1 × 10^6^ viable cells/mL, while the cocultures consisted of a 2:1 ratio (e.g. 2 × 10^6^ viable cells/mL:1 × 10^6^ viable cells/mL) S. cerevisiae:L. thermotolerans. The bars represent means of biological triplicates with whiskers indicating standard error of the mean respectively.


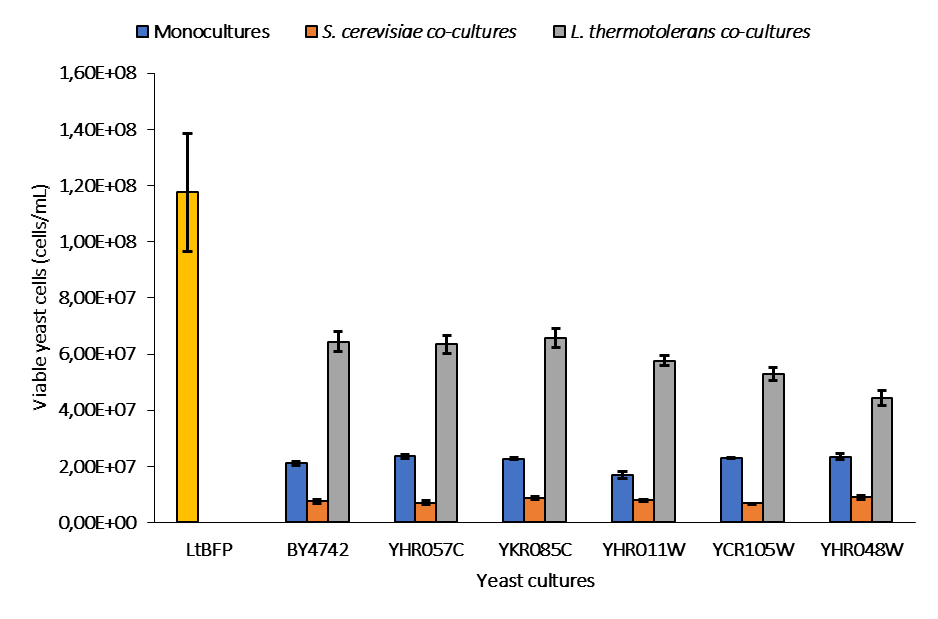


**C**


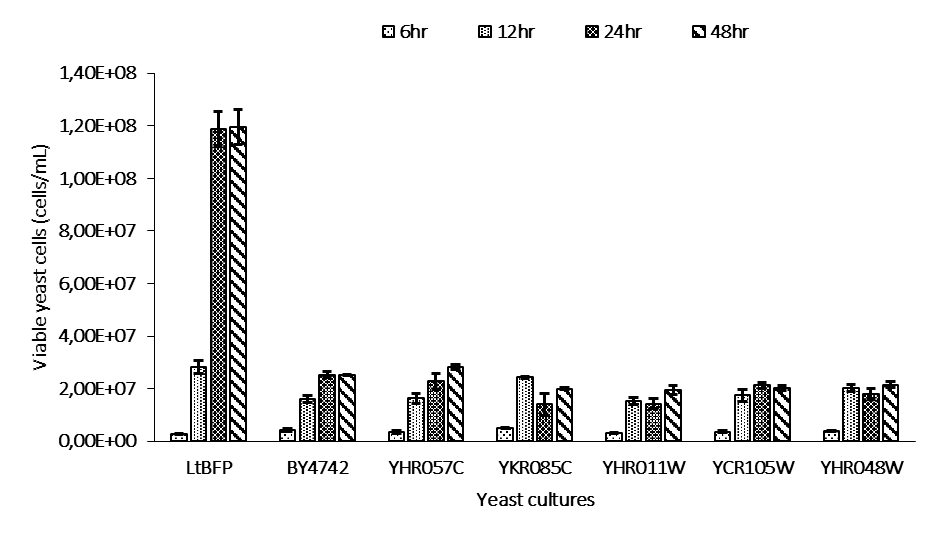


**A**

**Figure S10** (A) Monocultures of S. cerevisiae BY4742 deletion mutants and wildtype, as well as L. thermotolerans control (“LtBFP”) cultured in ISA-SGM following the same procedures described in Figure S9A. Growth of yeast cultures were monitored for 48-h, with samples taken at 6-, 12-, 24- and 48-h timepoints. Bars represent means of biological replicates with standard error of the mean indicated in each case.

**Figure S9 (continued)** (C) S. cerevisiae monocultures were inoculated into ISA-SGM at an initial cell density of 2 × 10^6^ viable cells/mL and L. thermotolerans control (“LtBFP” yellow bar) was inoculated at 0.5 × 10^6^ viable cells/mL, with cocultures inoculated in a 2:0.5 ratio (e.g. 2 × 10^6^ viable cells/mL:0.5 × 10^6^ viable cells/mL) S. cerevisiae:L. thermotolerans.

**C**

**B**

**Figure S10 (continued)** Viable yeast cell numbers of (B) S. cerevisiae BY4742 deletion mutants and wildtype and (C) L. thermotolerans (“LtBFP”) that were cultured in mixed fermentations in ISA-SGM following the same procedures described in Figure S9A. Growth of yeast cultures were monitored by collecting samples at 6-, 12-, 24- and 48-h timepoints. Means of biological replicates with standard error of the mean are shown by bars with whiskers.

**Figure S11** (A) Plot depicting viable yeast cell numbers (y-axis) for additional S. cerevisiae BY4742 deletion mutants in the upregulated gene list (x-axis) that were revived and screened by coculturing with L. thermotolerans (“LtBFP” yellow bar) control. The conditions for the screening were the same as mentioned for Figure S7B, however, these results were obtained after 12-h of culturing in ISA-SGM with the same initial inoculation ratios applied as mentioned in Figure S9(C). Monocultures and cocultures of S. cerevisiae BY4742 wildtype and L. thermotolerans served as controls for the experiments and references for phenotypic profile comparisons. Cell numbers for monocultures (blue bars) and cocultures of S. cerevisiae (“S. cerevisiae co-cultures”, orange bars) and L. thermotolerans (“L. thermotolerans co-cultures”, grey bars) in mixed fermentations are shown. The data represent a single biological replicate in each case.

**Figure S11 (continued)** (B) Plot depicting viable yeast cell numbers (y-axis) for additional S. cerevisiae BY4742 deletion mutants in the upregulated gene list (x-axis) that were revived and screened by coculturing with L. thermotolerans (“LtBFP” yellow bar) control. The culturing conditions and sampling times are the same as outlined in (A). The data represent a single biological replicate in each case, apart from deletion mutant cultures of “YMR169C”, “YKR053C” and “YLR382C” which represent the mean of two biological replicates with standard error of the mean indicated for each dataset.

**Figure S12** (A) Plot depicting viable yeast cell numbers (y-axis) for last additional representatives of S. cerevisiae BY4742 deletion mutants in the upregulated gene list as well as representatives corresponding to genes from the downregulated gene list obtained from analysis (x-axis) after revival and screening (same as mentioned for Figure S7B) in cocultures with L. thermotolerans (“LtBFP” yellow bar, control) for 12-h in ISA-SGM with initial inoculation ratios as mentioned in Figure S9(C). Monocultures and cocultures of S. cerevisiae BY4742 wildtype and L. thermotolerans were used as controls and references for phenotypic profile comparisons. Cell numbers for monocultures (blue bars) and cocultures of S. cerevisiae (“S. cerevisiae co-cultures”, orange bars) and L. thermotolerans (“L. thermotolerans co-cultures”, grey bars) in mixed fermentations are shown, with data representing single biological replicates in each case.

**B**

**Figure S12 (continued)** (B) Plot depicting viable yeast cell numbers (y-axis) for additional representatives of S. cerevisiae BY4742 deletion mutants corresponding to genes from the downregulated gene list (x-axis) in cocultures with L. thermotolerans (“LtBFP” yellow bar, control) under conditions mentioned in (A). Cell numbers for monocultures (blue bars) and cocultures of S. cerevisiae (“S. cerevisiae co-cultures”, orange bars) and L. thermotolerans (“L. thermotolerans co-cultures”, grey bars) in mixed fermentations are shown with monocultures and cocultures S. cerevisiae BY4742 wildtype and L. thermotolerans as serving as controls and phenotypic references. Data represent single biological replicates respectively.

**C**

**Figure S12 (continued)** (C) Viable yeast cell numbers (y-axis) for additional representatives of S. cerevisiae BY4742 deletion mutants corresponding to genes from the downregulated gene list (x-axis) cocultured with L. thermotolerans (“LtBFP” yellow bar, control) as mentioned in (B). Screening was conducted under conditions mentioned in (A), data representing single biological replicates for monocultures (blue bars) and cocultures of S. cerevisiae (“S. cerevisiae co-cultures”, orange bars) and L. thermotolerans (“L. thermotolerans co-cultures”, grey bars) in mixed fermentations are shown.

**Figure S13** Agarose gel (1% w/vol) depicting PCR products of transformants of S. cerevisiae VIN13 (lanes 3-9) and BY4741 wildtype (lane 12) that were subjected to CRISPR-Cas9 mediated FIT2 gene deletion. A FastRuler Low Range (50 to 1500 bp) DNA Ladder was used for size estimations (lanes 1 and 11). The positive control (lane 10) was VIN13 wildtype and a “no template” negative amplification control was also included (lane 2). The VIN13 control (lane 10) had a larger amplification fragment (ca. 1500 bp) than the transformants (ca. 850 bp, lanes 3-9 and lane 12) indicating that the FIT2 gene (462 bp) was successfully deleted in the transformants.


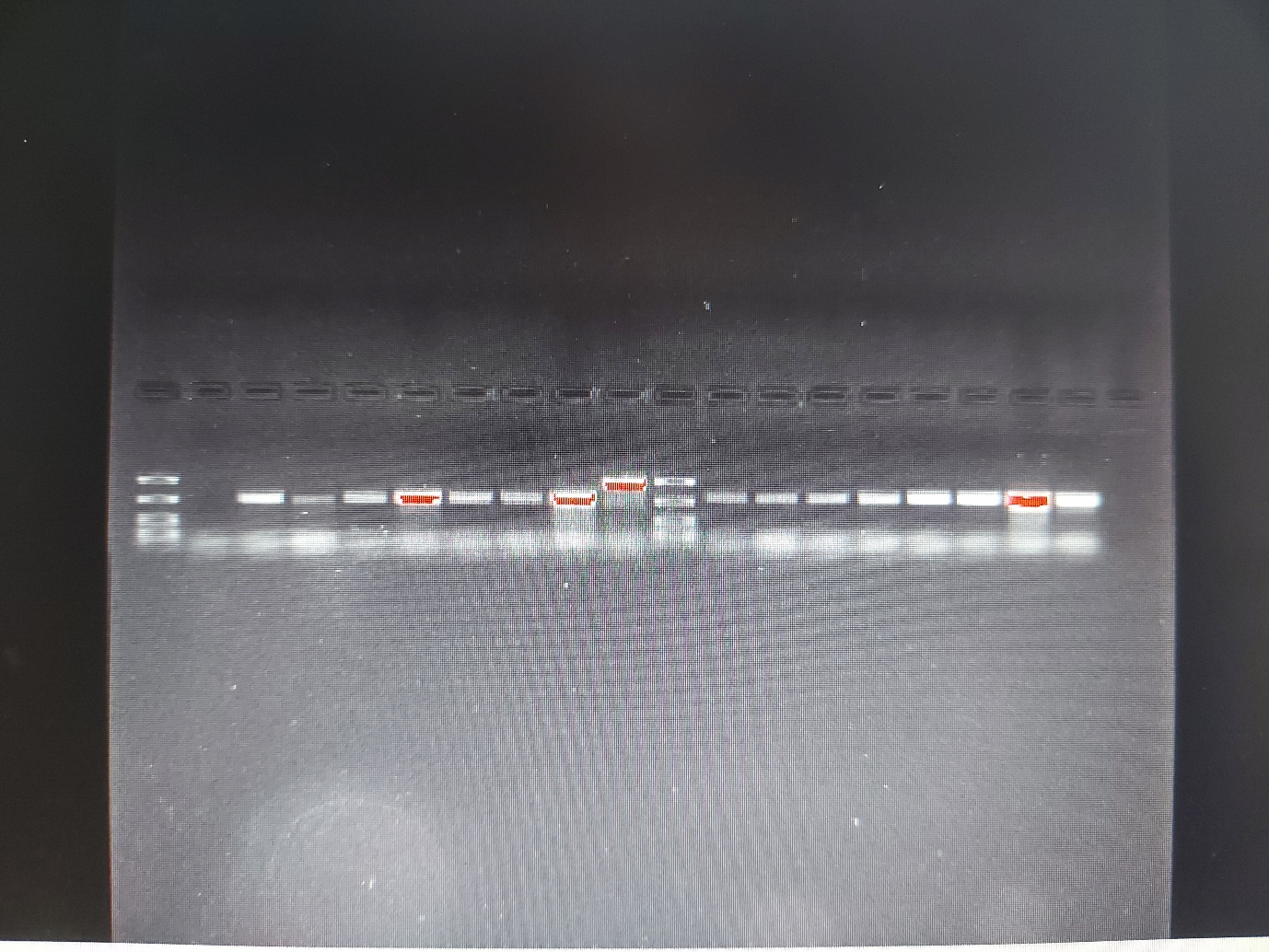


**1**

**2**

**3**

**4**

**5**

**6**

**7**

**8**

**9**

**10**

**11**

**12**

**1500 bp**

**850 bp**

**Figure S12 (continued)** (D) Viable yeast cell numbers (y-axis) for final additional representatives of S. cerevisiae BY4742 deletion mutants related to genes from the downregulated gene list (x-axis) cocultured with L. thermotolerans (“LtBFP” yellow bar, control) mentioned in (B). Screening was conducted under conditions described in (A). Data represent single biological replicates for monocultures (blue bars) and cocultures of S. cerevisiae (“S. cerevisiae co-cultures”, orange bars) and L. thermotolerans (“L. thermotolerans co-cultures”, grey bars) in mixed fermentations.

**D**

**Figure** **S14** Plot depicting differences in viable yeast cell numbers (y-axis) for deletion mutant strain representatives and wildtype S. cerevisiae BY4742 (x-axis) in coculture with L. thermotolerans under the same conditions mentioned in Figure 4A. Cell numbers for monocultures (blue bars) and mixed cultures of S. cerevisiae (orange bars) and L. thermotolerans (grey bars) are shown, with S. cerevisiae deletion mutant strains that were tested in coculture with L. thermotolerans indicated by the deleted gene names e.g. ((A) PMS1 to STL1 and (B) RTC3 to MSS1) that were compared to coculture phenotype profiles of the wildtype strain (e.g. BY4742). The data represent means of biological replicates with standard error of the mean, while significant differences (p-value < 0.05) are indicated by the lower-case letters as mentioned in Figure 4A.

**Figure S14 (continued)** Plots showing differences in viable yeast cell numbers (y-axis) for deletion mutants of the wildtype S. cerevisiae BY4742/BY4741 strains (x-axis) in coculture with L. thermotolerans under the same conditions mentioned in Figure 4A. Monocultures (blue bars) and mixed cultures of S. cerevisiae (orange bars) and L. thermotolerans (grey bars) are displayed with deleted gene names (e.g. (C) XLY2 to ORM2) and (D) MET17 to MET3) representing cultures of S. cerevisiae deletion mutants tested in coculture with L. thermotolerans that were compared to cocultures of the wildtype BY4741 strain. Data represent means of three biological repeats with standard error of the mean respectively. Significant differences (p-value < 0.05) are indicated by lower-case letters as mentioned in Figure 4A.


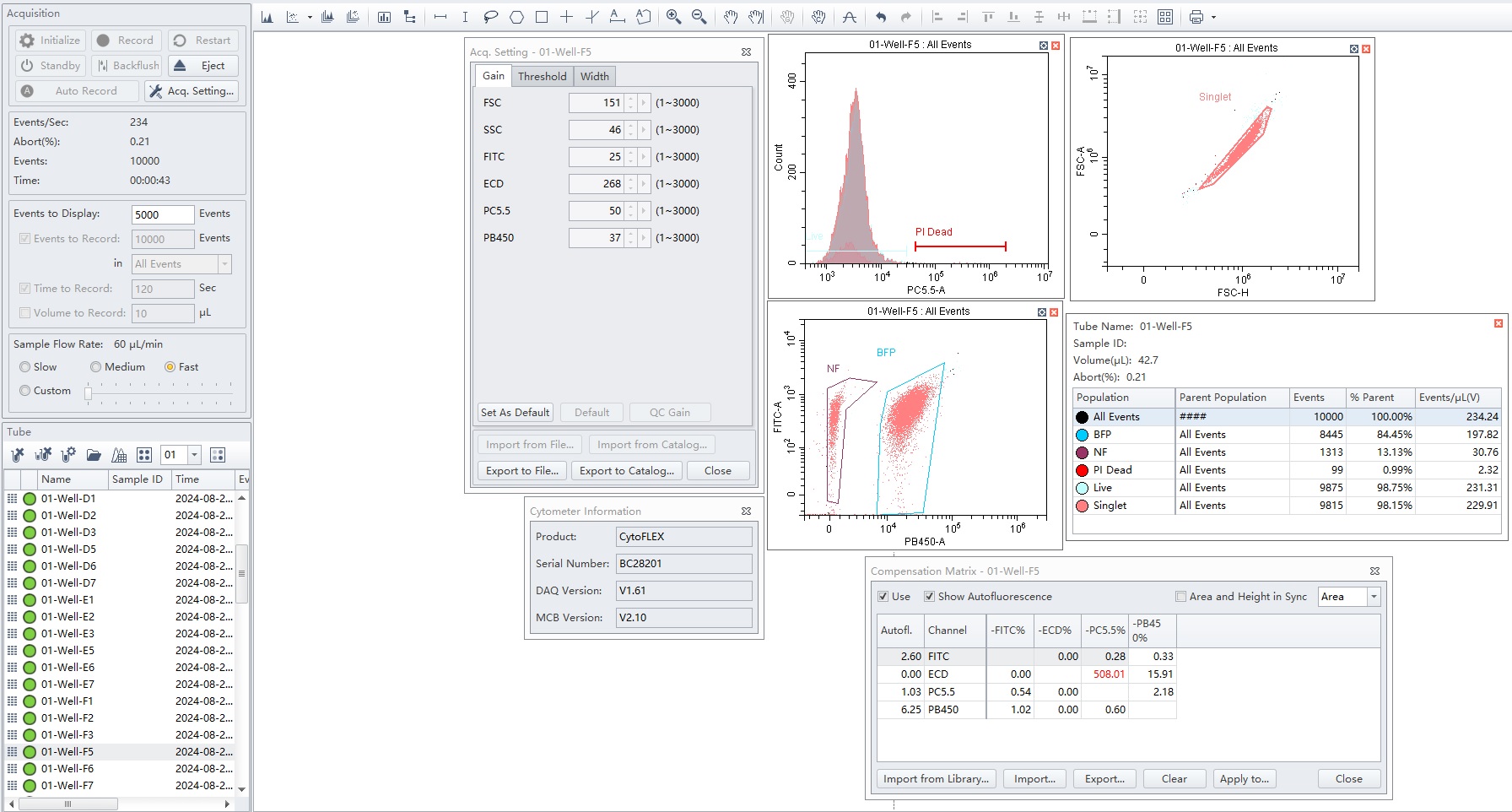


**Figure 15** Illustration of the gating strategy, compensation and channels that were used for flow cytometry measurements of cocultures during screening.

Supplementary Text S1:

**Text S2** CLUSTAL multiple sequence alignment (MSA) output using MUSCLE (version 3.8) for sequences obtained from amplification products of representatives of *S. cerevisiae* VIN13 (“VIN13_Transformant_1/2/6/7”)and BY4741 (“BY4741_Transformant_9”) after CRISPR-Cas9 mediated *FIT2* gene deletion. *S. cerevisiae* VIN13 wildtype (“VIN13_CONTROL”) was used as a control and the reference sequence for *FIT2* coding sequence and flanking regions (“FIT2_FLANKING_AND_CDS”) was obtained for *S. cerevisiae* S288C from the *Saccharomyces* genome database (SGD). The region containing the coding sequence (CDS) of *FIT2* is highlighted in the sequence alignment below, which is absent in transformants and present in the *S. cerevisiae* S288C reference and *S. cerevisiae* VIN13 wildtype.

VIN13_Transformant_6 ------------------------------------------------------------

VIN13_Transformant_2 ------------------------------------------------------------

VIN13_Transformant_1 ------------------------------------------------------------

VIN13_CONTROL ------------------------------------------------------------

BY4741_Transformant_9 ------------------------------------------------------------

FIT2_FLANKING_AND_CDS ACGCAAGACAACAGGCAAAATAATTTCGTTTCTCAGTACCGAAATGACGAAATATACTGA

VIN13_Transformant_7 ------------------------------------------------------------

VIN13_Transformant_6 ------------------------------------------------------------

VIN13_Transformant_2 ------------------------------------------------------------

VIN13_Transformant_1 ------------------------------------------------------------

VIN13_CONTROL ------------------------------------------------------------

BY4741_Transformant_9 ------------------------------------------------------------

FIT2_FLANKING_AND_CDS GGCAAATGCGATCATCATGCCTTTGCGCCAAGAAACTCCCTTGTGAAGAACTTCAAACCG

VIN13_Transformant_7 ------------------------------------------------------------

VIN13_Transformant_6 ------------------------------------------------------------

VIN13_Transformant_2 ------------------------------------------------------------

VIN13_Transformant_1 ------------------------------------------------------------

VIN13_CONTROL ------------------------------------------------------------

BY4741_Transformant_9 ------------------------------------------------------------

FIT2_FLANKING_AND_CDS AAATGGGAAAACTTTGAGTTATTGACAGGGAATACGGAGGGGAAGATCACACTTAAATCC

VIN13_Transformant_7 ------------------------------------------------------------

VIN13_Transformant_6 ------------------------------------------------------------

VIN13_Transformant_2 ------------------------------------------------------------

VIN13_Transformant_1 ------------------------------------------------------------

VIN13_CONTROL ------------------------------------------------------------

BY4741_Transformant_9 ------------------------------------------------------------

FIT2_FLANKING_AND_CDS GTATGAGCCGCGCACATAATGGTATTCAAATACACAAGAACATTCATGAGCTATTTTTCA

VIN13_Transformant_7 ------------------------------------------------------------

VIN13_Transformant_6 ------------------------------------------------------------

VIN13_Transformant_2 ------------------------------------------------------------

VIN13_Transformant_1 ------------------------------------------------------------

VIN13_CONTROL ------------------------------------------------------------

BY4741_Transformant_9 ------------------------------------------------------------

FIT2_FLANKING_AND_CDS TCCGTGCAAACGAATTTACTACAATTGGACCAGAGGGCACCATAACTGGAGACTTTGCTA

VIN13_Transformant_7 ------------------------------------------------------------

VIN13_Transformant_6 ------------------------------------------------------------

VIN13_Transformant_2 ------------------------------------------------------------

VIN13_Transformant_1 ------------------------------------------------------------

VIN13_CONTROL ------------------------------------------------------------

BY4741_Transformant_9 ------------------------------------------------------------

FIT2_FLANKING_AND_CDS CTGACTCAACGTTGATGATGCGAGTAGTGGGTGTACTGTGATTTGCTCATTTTTTTTTTT

VIN13_Transformant_7 ------------------------------------------------------------

VIN13_Transformant_6 ------------------------------------------------------------

VIN13_Transformant_2 ------------------------------------------------------------

VIN13_Transformant_1 ------------------------------------------------------------

VIN13_CONTROL ------------------------------------------------------------

BY4741_Transformant_9 ------------------------------------------------------------

FIT2_FLANKING_AND_CDS ATAGAAAGATTCGATTAATGAAAGTCACAGGAGACATTTTTACATAGACATTCCGTATAT

VIN13_Transformant_7 ------------------------------------------------------------

VIN13_Transformant_6 ------------------------------------------------------------

VIN13_Transformant_2 ------------------------------------------------------------

VIN13_Transformant_1 ------------------------------------------------------------

VIN13_CONTROL ------------------------------------------------------------

BY4741_Transformant_9 ------------------------------------------------------------

FIT2_FLANKING_AND_CDS GTTGCGGGTATCGCGGATGCGGATTAGTGATGCCTTTAACTACATTTCATAGATTTCTGT

VIN13_Transformant_7 ------------------------------------------------------------

VIN13_Transformant_6 ------------------------------------------------------------

VIN13_Transformant_2 ------------------------------------------------------------

VIN13_Transformant_1 ------------------------------------------------------------

VIN13_CONTROL ------------------------------------------------------------

BY4741_Transformant_9 ------------------------------------------------------------

FIT2_FLANKING_AND_CDS ATACCAATTGAAATGAGTGAAGTAAGCTCCTACAGTGAAATATCTGGGTGCTACTGACGC

VIN13_Transformant_7 ------------------------------------------------------------

VIN13_Transformant_6 ------------------------------------------------------------

VIN13_Transformant_2 ------------------------------------------------------------

VIN13_Transformant_1 ------------------------------------------------------------

VIN13_CONTROL ------------------------------------------------------------

BY4741_Transformant_9 ------------------------------------------------------------

FIT2_FLANKING_AND_CDS CAAGCCCTACAGCGATCGGAATGCGGGAACGGAAGTTAACGGGGCTTCCAGAACGGCGGA

VIN13_Transformant_7 ------------------------------------------------------------

VIN13_Transformant_6 -----------------------------------------------------ATGACCC

VIN13_Transformant_2 ------------------------------------------------------------

VIN13_Transformant_1 ------------------------------------------------------------

VIN13_CONTROL ---------------------------------------------TACTTAGAATGACCC

BY4741_Transformant_9 ---------------------------------------------TACTTAGAATGACCC

FIT2_FLANKING_AND_CDS AGCGAATTGAACGAGGACGGCAAACAAAAACACCCAAAATTTCATTACTTAGAATGACCC

VIN13_Transformant_7 ------------------------------------------------------------

VIN13_Transformant_6 TCAAGAGCAGGGTGCAATTTATCAAGCGATCATTGAACTAACTAAGTTCATATCCTGTAT

VIN13_Transformant_2 --------------CAATTTATCGAGCGATCATTGAACTAACTAAGTTCATATCCTGTAT

VIN13_Transformant_1 --------AGGGTGCAATTTATCAAGCGATCATTGAACTAACTAAGTTCATATCCTGTAT

VIN13_CONTROL TCAAGAGCAGGGTGCAATTTATCAAGCGATCATTGAACTAACTAAGTTCATATCCTGTAT

BY4741_Transformant_9 TCAAGAGCAGGGTGCAATTTATCAAGCGATCATTGAACTAACTAAGTTCATATCCTGTAT

FIT2_FLANKING_AND_CDS TCAAGAGCAGGGTGCAATTTATCAAGCGATCATTGAACTAACTAAGTTCATATCCTGTAT

VIN13_Transformant_7 -----AGCAGGGTGCAATTTATCAAGCGATCATTGAACTAACTAAGTTCATATCCTGTAT

********* ************************************

VIN13_Transformant_6 AGGATTTAAAACAATGCACCCTAAGTTCAAATGCACCCCCCCTCGCCCCGCAGCGGACCC

VIN13_Transformant_2 AGGATTTAAAACAATGCACCCTAAGTTCAAATGCACCCCCCCTCGCCCCGCAGCGGACCC

VIN13_Transformant_1 AGGATTTAAAACAATGCACCCTAAGTTCAAATGCACCCCCCCTCGCCCCGCAGCGGACCC

VIN13_CONTROL AGGATTTAAAACAATGCACCCTAAGTTCAAATGCACCCCCCCTCGCCCCGCAGCGGACCC

BY4741_Transformant_9 AGGATTTAAAACAATGCACCCTAAGTTCAAATGCACCCCCCCTCGCCCCGCAGCGGACCC

FIT2_FLANKING_AND_CDS AGGATTTAAAACAATGCACCCTAAGTTCAAATGCACCCCCCCTCGCCCCGCAGCGGACCC

VIN13_Transformant_7 AGGATTTAAAACAATGCACCCTAAGTTCAAATGCACCCCCCCTCGCCCCGCAGCGGACCC

************************************************************

VIN13_Transformant_6 TTGAACAGAGAACTGTTTCGAGGTTCACCCAATTGGATCACTTGTATAATTTGTAATCGA

VIN13_Transformant_2 TTGAACAGAGAACTGTTTCGAGGTTCACCCAATTGGATCACTTGTATAATTTGTAATCGA

VIN13_Transformant_1 TTGAACAGAGAACTGTTTCGAGGTTCACCCAATTGGATCACTTGTATAATTTGTAATCGA

VIN13_CONTROL TTGAACAGAGAACTGTTTCGAGGTTCACCCAATTGGATCACTTGTATAATTTGTAATCGA

BY4741_Transformant_9 TTGAACAGAGAACTGTTTCGAGGTTCACCCAATTGGATCACTTGTATAATTTGTAATCGA

FIT2_FLANKING_AND_CDS TTGAACAGAGAACTGTTTCGAGGTTCACCCAATTGGATCACTTGTATAATTTGTAATCGA

VIN13_Transformant_7 TTGAACAGAGAACTGTTTCGAGGTTCACCCAATTGGATCACTTGTATAATTTGTAATCGA

************************************************************

VIN13_Transformant_6 GTTCGGATAAAATGTATACGAATCTAACTGGGTGCAGTATAAATAGCATTTTATATTACC

VIN13_Transformant_2 GTTCGGATAAGATGTATACGAATCTAACTGGGTGCAGTATAATTAGCATTTTATATTACC

VIN13_Transformant_1 GTTCGGATAAGATGTATACGAATCTAACTGGGTGCAGTATAATTAGCATTTTATATTACC

VIN13_CONTROL GTTCGGATAAAATGTATACGAATCTAACTGGGTGCAGTATAATTAGCATTTTATATTACC

BY4741_Transformant_9 GTTCGGATAAGATGTATACGAATCTAACTGGGTGCAGTATAATTAGCATTTTATATTACC

FIT2_FLANKING_AND_CDS GTTCGGATAAGATGTATACGAATCTAACTGGGTGCAGTATAATTAGCATTTTATATTACC

VIN13_Transformant_7 GTTCGGATAAGATGTATACGAATCTAACTGGGTGCAGTATAATTAGCATTTTATATTACC

********** ******************************* *****************

VIN13_Transformant_6 TAACAATATATGTATAAAACAGGAGTGTGTGAGTGCTCCAGGCAGAATTTTACGGTCCTT

VIN13_Transformant_2 TAGCAATATATGTATAAAACAGGAATGTGTGCGTGCTTCAGGCAGAATTTTACGGTCCTT

VIN13_Transformant_1 TAGCAATATATGTATAAAACAGGAATGTGTGCGTGCTTCAGGCAGAATTTTACGGTCCTT

VIN13_CONTROL TAGCAATATATGTATAAAACAGGAATGTGTGCGTGCTTCAGGCAGAATTTTACGGTCCTT

BY4741_Transformant_9 TAGCAATATATGTATAAAACAGGAATGTGTGCGTGCTTCAGGCAGAATTTTACGGTCCTT

FIT2_FLANKING_AND_CDS TAGCAATATATGTATAAAACAGGAATGTGTGCGTGCTTCAGGCAGAATTTTACGGTCCTT

VIN13_Transformant_7 TAGCAATATATGTATAAAACAGGAATGTGTGCGTGCTTCAGGCAGAATTTTACGGTCCTT

** ********************* ****** ***** **********************

VIN13_Transformant_6 GTAAAAAAGTCTATCATAAAGCCATCACAYAACAATAA----------------------

VIN13_Transformant_2 GTAAAAAAGTCTATCATAAAGCCATCACAAAACAATAA----------------------

VIN13_Transformant_1 GTAAAAAAGTCTATCATAAAGCCATCACAAAACAATAA----------------------

VIN13_CONTROL GTAAAAAAGTCTATCATAAAGCCATCACAAAACAATAATAATGAAATTCTCAACTATTTT

BY4741_Transformant_9 GTAAAAAAGTCTATCATAAAGCCATCACAAAACAATAA----------------------

FIT2_FLANKING_AND_CDS GTAAAAAAGTCTATCATAAAGCCATCACAAAACAATAATAATGAAATTCTCAACTATTTT

VIN13_Transformant_7 GTAAAAAAGTCTATCATAAAGCCATCACAAAACAATAA----------------------

***************************** ********

VIN13_Transformant_6 ------------------------------------------------------------

VIN13_Transformant_2 ------------------------------------------------------------

VIN13_Transformant_1 ------------------------------------------------------------

VIN13_CONTROL CGGAGCTACTACAGTTATGACTGCCGTCTCGGCAGCAGCTGTGTCGAGTGTAATGACCAC

BY4741_Transformant_9 ------------------------------------------------------------

FIT2_FLANKING_AND_CDS CGGAGCTACTACAGTTATGACTGCCGTCTCGGCAGCAGCTGTGTCGAGTGTAATGACCAC

VIN13_Transformant_7 ------------------------------------------------------------

VIN13_Transformant_6 ------------------------------------------------------------

VIN13_Transformant_2 ------------------------------------------------------------

VIN13_Transformant_1 ------------------------------------------------------------

VIN13_CONTROL TAAGACTATTACTGCTACTAACGGTAATAACGTTTACACTAAGGTCGTTACCGACACCGC

BY4741_Transformant_9 ------------------------------------------------------------

FIT2_FLANKING_AND_CDS TAAGACTATTACTGCTACTAACGGTAATAACGTTTACACTAAGGTCGTTACCGACACCGC

VIN13_Transformant_7 ------------------------------------------------------------

VIN13_Transformant_6 ------------------------------------------------------------

VIN13_Transformant_2 ------------------------------------------------------------

VIN13_Transformant_1 ------------------------------------------------------------

VIN13_CONTROL TGACCCTATCATTAGTTACAGTACCACTAGAACTGTCGTTGTCAGTAATAGTGATGCTAC

BY4741_Transformant_9 ------------------------------------------------------------

FIT2_FLANKING_AND_CDS TGACCCTATCATTAGTTACAGTACCACTAGAACTGTCGTTGTCAGTAATAGTGATGCTAC

VIN13_Transformant_7 ------------------------------------------------------------

VIN13_Transformant_6 ------------------------------------------------------------

VIN13_Transformant_2 ------------------------------------------------------------

VIN13_Transformant_1 ------------------------------------------------------------

VIN13_CONTROL TTACACAAAGGTTTGTCACCGAAGGACCAGATACCACCTCTGAAAAGAGTACAACAAAGA

BY4741_Transformant_9 ------------------------------------------------------------

FIT2_FLANKING_AND_CDS TTACACAAAGG-TTGTCACCGAAGGACCAGATACCACCTCTGAAAAGAGTACAACAAAGA

VIN13_Transformant_7 ------------------------------------------------------------

VIN13_Transformant_6 ------------------------------------------------------------

VIN13_Transformant_2 ------------------------------------------------------------

VIN13_Transformant_1 ------------------------------------------------------------

VIN13_CONTROL CACTTACTTTGACAAACGGTTCAGGTTCATCAACCAACCTTTACACCAAGACCGTCACTC

BY4741_Transformant_9 ------------------------------------------------------------

FIT2_FLANKING_AND_CDS CACTTACTTTGACAAACGGTTCAGGTTCATCAACCAACCTTTACACCAAGACCGTCACTC

VIN13_Transformant_7 ------------------------------------------------------------

VIN13_Transformant_6 ------------------------------------------------------------

VIN13_Transformant_2 ------------------------------------------------------------

VIN13_Transformant_1 ------------------------------------------------------------

VIN13_CONTROL AAGCCGTCGAATCATCTACATCCTCCTCATCCTCCTCATCCTCCTCCTCCTCTTCTGCCT

BY4741_Transformant_9 ------------------------------------------------------------

FIT2_FLANKING_AND_CDS AAGCCGTCGAATCATCTACATCCTCCTCATCCTCCTCATCCTCCTCCTCCTCTTCTGCCT

VIN13_Transformant_7 ------------------------------------------------------------

VIN13_Transformant_6 ------------------------------------------------------------

VIN13_Transformant_2 ------------------------------------------------------------

VIN13_Transformant_1 ------------------------------------------------------------

VIN13_CONTROL CTTCTTCTGGTGCTGCTCCTGCTGCATTCCAAGGAGCAAGTGTCGGTGCATTGGCCCTTG

BY4741_Transformant_9 ------------------------------------------------------------

FIT2_FLANKING_AND_CDS CTTCTTCTGGTGCTGCTCCTGCTGCATTCCAAGGAGCAAGTGTCGGTGCATTGGCCCTTG

VIN13_Transformant_7 ------------------------------------------------------------

VIN13_Transformant_6 ---------------------TAGCTCGGTGACAAACAAGAAAAAAGCTATTAGTTATCG

VIN13_Transformant_2 ---------------------TAGCTCGGTTTCAAACCAGAAAAAAGCTATTAGTTATCG

VIN13_Transformant_1 ---------------------TAGCTCGGTTTCAAACCAGAAAAAAGCTATTAGTTATCG

VIN13_CONTROL GTTTGATTTCTTACCTATTATAAGCTCGGTTTCAAACCAGAAAAAAGCTATTAGTTATCG

BY4741_Transformant_9 ---------------------AAGCTCGGTTTCAAACCAGAAAAAAGCTATTAGTTATCG

FIT2_FLANKING_AND_CDS GTTTGATTTCTTACCTATTATAAGCTCGGTTTCAAACCAGAAAAAAGCTATTAGTTATCG

VIN13_Transformant_7 ---------------------AAGCTCGGTTTCAAACCAGAAAAAAGCTATTAGTTATCG

******** ***** **********************

VIN13_Transformant_6 TGTATAGCATATTTATAACTTATCTATGAAAAGCTAATATAATTGTCTTTCTCCTTCAAA

VIN13_Transformant_2 TGTATAGCATATTTATAACTTATCTATGAAAAGCTAATATAATTGTCTTTCTCCTTCAAA

VIN13_Transformant_1 TGTATAGCATATTTATAACTTATCTATGAAAAGCTAATATAATTGTCTTTCTCCTTCAAA

VIN13_CONTROL TGTATAGCATATTTATAACTTATCTATGAAAAGCTAATATAATTGTCTTTCTCCTTCAAA

BY4741_Transformant_9 TGTATAGCATATTTATAACTTATCTATGAAAAGCTAATATAATTGTCTTTCTCCTTCAAA

FIT2_FLANKING_AND_CDS TGTATAGCATATTTATAACTTATCTATGAAAAGCTAATATAATTGTCTTTCTCCTTCAAA

VIN13_Transformant_7 TGTATAGCATATTTATAACTTATCTATGAAAAGCTAATATAATTGTCTTTCTCCTTCAAA

************************************************************

VIN13_Transformant_6 AGGCTAGGAAAAAGGACTTTTTTTCATTACATTTCTCAGTGACTTTAATGTAGCAGTTTT

VIN13_Transformant_2 AGGCTAGTAAAAAGGACTTTTTTTCATTACGTTTCTCAGTGACTTTAATGTAGCAGTTTT

VIN13_Transformant_1 AGGCTAGTAAAAAGGACTTTTTTTCATTACGTTTCTCAGTGACTTTAATGTAGCAGTTTT

VIN13_CONTROL AGGCTAGTAAAAAGGACTTTTTTTCATTACGTTTCTCAGTGACTTTAATGTAGCAGTTTT

BY4741_Transformant_9 AGGCTAGTAAAAAGGACTTTTTTTCATTACGTTTCTCAGTGACTTTAATGTAGCAGTTTT

FIT2_FLANKING_AND_CDS AGGCTAGTAAAAAGGACTTTTTTTCATTACGTTTCTCAGTGACTTCAATGTAGCAGTTTT

VIN13_Transformant_7 AGGCTAGTAAAAAGGACTTTTTTTCATTACGTTTCTCAGTGACTTCAATGTAGCAGTTTT

******* ********************** ************** **************

VIN13_Transformant_6 TCGTTTCATCCTCCTT-TCGGATTATTATTATTGTTATTATTATTATTATCATTACTTTT

VIN13_Transformant_2 TCGTTTCATCATCATCATCGTATTATTATTTTTGTTATTATTATTATTATCATTACTTTT

VIN13_Transformant_1 TCGTTTCATCATCATCATCGTATTATTATTATTGTTATTATTATTATTATCATTACTTTT

VIN13_CONTROL TCGTTTCATCATCATCATCGTATTATTATTATTGTTATTATTATTATTATCATTACTTTT

BY4741_Transformant_9 TCGTTTCATCATCATCATCGTATTATTATTATTGTTATTATTATTATTATCATTACTTTT

FIT2_FLANKING_AND_CDS TCGTTTCATCATCATCATCGTATTATTATTATTGTTATTATTATTATTATCATTACTTTT

VIN13_Transformant_7 TCGTTTCATCATCATCATCGTATTATTATTATTGTTATTATTATTATTATCATTACTTTT

********** ** * *** ********* *****************************

VIN13_Transformant_6 ATTA--------------------------------------------------------

VIN13_Transformant_2 ATTAATATTAACTATTTTTTTAGTATGATTTCGGGTATATTTATTTTAATTAGATACTTT

VIN13_Transformant_1 ATTAATATTAACTATTTTTTTAGTATGATTTCGGGTATATTTATTTTAATTAGATACTTT

VIN13_CONTROL ATTAATATTAACTATTTTTTTAGTATGATTTCGGGTATATTTATTTTAATTAGATACTTT

BY4741_Transformant_9 ATTAATATTAACTATTTTTTTAGTATGATTTCGGGTATATTTATTTTAATTAGATACTTT

FIT2_FLANKING_AND_CDS ATTAATATTAACTATTTTTTTAGTATGATTTCGGGTATATTTATTTTAATTAGATACTTT

VIN13_Transformant_7 ATTAATATTAACTATTTTTTTAGTATGATTTCGGGTATATTTATTTTAATTAGATACTTT

****

VIN13_Transformant_6 ------------------------------------------------------------

VIN13_Transformant_2 TAACCGTTTCAAACAATTAAAGGAAGACCAACGAACTTGAAACAGTATATATACATACAT

VIN13_Transformant_1 TAACCGTTTCAAACAATTAAAGGAAGACCAACGAACTTGAAACAGTATATATACATACAT

VIN13_CONTROL TAACCGTTTCAAACAATTAAAGGAAGACCAACGAACTTGAAACAGTATATATACATACAT

BY4741_Transformant_9 TAACCGTTTCAAACAATTAAAGGAAGACCAACGAACTTGAAACAGTATATATACATACAT

FIT2_FLANKING_AND_CDS TAACCGTTTCAAACAATTAAAGGAAGACCAACGAACTTGAAACAGTATATATACATACAT

VIN13_Transformant_7 TAACCGTTTCAAACAATTAAAGGAAGACCAACGAACTTGAAACAGTATATATACATACAT

VIN13_Transformant_6 ------------------------------------------------------------

VIN13_Transformant_2 ACTTAATGTGTGTTATTAAAATTTTTTTATTCTAACATAACTTCGAAAAAAGGCCAAGCC

VIN13_Transformant_1 ACTTAATGTGTGTTATTAAAATTTTTTTATTCTAACATAACTTCGAAAAAAGGCCAAGCC

VIN13_CONTROL ACTTAATGTGTGTTATTAAAATTTTTTTATTCTAACATAACTTCGAAAAAAGGCCAAGCC

BY4741_Transformant_9 ACTTAATGTGTGTTATTAAAATTTTTTTATTCTAACATAACTTCGAAAAAAGGCCAAGCC

FIT2_FLANKING_AND_CDS ACTTAATGTGTGTTATTAAAATTTTTTTATTCTAACATAACTTCGAAAAAAGGCCAAGCC

VIN13_Transformant_7 ACTTAATGTGTGTTATTAAAATTTTTTTATTCTAACATAACTTCGAAAAAAGGCCAAGCC

VIN13_Transformant_6 ------------------------------------------------------------

VIN13_Transformant_2 GCAAGGTGCTTCATAAAAATCGGATATCCCGC----------------------------

VIN13_Transformant_1 GCAAGGTGCTTCATAAAAATCGGATATCCCGCATG-------------------------

VIN13_CONTROL GCAAGGTGCTTCATAAAAATCGGATATCCCGCA---------------------------

BY4741_Transformant_9 GCAAGGTGCTTCATAAAAATCGGATATCCCGCATGATTATTCACATATC-----------

FIT2_FLANKING_AND_CDS GCAAGGTGCTTCATAAAAATCGGATATCCCGCATGATTATTCACATATCATTTACAATAA

VIN13_Transformant_7 GCAAGGTGCTTCATAAAAATCGGATATCCCGCATG-------------------------

VIN13_Transformant_6 ------------------------------------------------------------

VIN13_Transformant_2 ------------------------------------------------------------

VIN13_Transformant_1 ------------------------------------------------------------

VIN13_CONTROL ------------------------------------------------------------

BY4741_Transformant_9 ------------------------------------------------------------

FIT2_FLANKING_AND_CDS CATGACGGCAGCAAGGGCAGCACCCATCAAACCAGTACCAGCGGTGATGGCATCAGCAGC

VIN13_Transformant_7 ------------------------------------------------------------

VIN13_Transformant_6 ------------------------------------------------------------

VIN13_Transformant_2 ------------------------------------------------------------

VIN13_Transformant_1 ------------------------------------------------------------

VIN13_CONTROL ------------------------------------------------------------

BY4741_Transformant_9 ------------------------------------------------------------

FIT2_FLANKING_AND_CDS ACCGGTGTAACTAGATAATTCAGCGGTGCTAGAGGATGTAGCAGAGGAAGAAGTTTCAGC

VIN13_Transformant_7 ------------------------------------------------------------

VIN13_Transformant_6 ------------------------------------------------------------

VIN13_Transformant_2 ------------------------------------------------------------

VIN13_Transformant_1 ------------------------------------------------------------

VIN13_CONTROL ------------------------------------------------------------

BY4741_Transformant_9 ------------------------------------------------------------

FIT2_FLANKING_AND_CDS AGTGGTGGTTGCAGTGGTTGAAGCAGAAGAGGTAGCAGCTTCTGAGCTGGTAGAAGTACT

VIN13_Transformant_7 ------------------------------------------------------------

VIN13_Transformant_6 ------------------------------------------------------------

VIN13_Transformant_2 ------------------------------------------------------------

VIN13_Transformant_1 ------------------------------------------------------------

VIN13_CONTROL ------------------------------------------------------------

BY4741_Transformant_9 ------------------------------------------------------------

FIT2_FLANKING_AND_CDS ACTTGGAGACCAGGTGTTGCTGCTGCCTTCACCAGTCCAGACAAAAGTAGCATCTTGGGT

VIN13_Transformant_7 ------------------------------------------------------------

VIN13_Transformant_6 ------------------------------------------------------------

VIN13_Transformant_2 ------------------------------------------------------------

VIN13_Transformant_1 ------------------------------------------------------------

VIN13_CONTROL ------------------------------------------------------------

BY4741_Transformant_9 ------------------------------------------------------------

FIT2_FLANKING_AND_CDS GACAGTCTTAGTGTAGACGTGACCGTTCTTGGTGGCAGTGATAGTGGTAGTGATACTAGA

VIN13_Transformant_7 ------------------------------------------------------------

VIN13_Transformant_6 ------------------------------------------------------------

VIN13_Transformant_2 ------------------------------------------------------------

VIN13_Transformant_1 ------------------------------------------------------------

VIN13_CONTROL ------------------------------------------------------------

BY4741_Transformant_9 ------------------------------------------------------------

FIT2_FLANKING_AND_CDS ACCAGAACCTTCATCAGCAGAAGAAGTCTCAGCAGCAGAAGAAGTCTCAGCAGCAGAAGT

VIN13_Transformant_7 ------------------------------------------------------------

VIN13_Transformant_6 ------------------------------------------------------------

VIN13_Transformant_2 ------------------------------------------------------------

VIN13_Transformant_1 ------------------------------------------------------------

VIN13_CONTROL ------------------------------------------------------------

BY4741_Transformant_9 ------------------------------------------------------------

FIT2_FLANKING_AND_CDS GGTAGCGGCAGCAGAAGTGGTAGCGGCAGCAGAGGTTTCGGCGGCAGAAGTTTCGGCGGC

VIN13_Transformant_7 ------------------------------------------------------------

VIN13_Transformant_6 ------------------------------------------------------------

VIN13_Transformant_2 ------------------------------------------------------------

VIN13_Transformant_1 ------------------------------------------------------------

VIN13_CONTROL ------------------------------------------------------------

BY4741_Transformant_9 ------------------------------------------------------------

FIT2_FLANKING_AND_CDS AGAAGATTCAGCGGCAGAAGTGCTGCTGGCGTAAGAGTCTTCACCACCCCAAACAAAAGT

VIN13_Transformant_7 ------------------------------------------------------------

VIN13_Transformant_6 ------------------------------------------------------------

VIN13_Transformant_2 ------------------------------------------------------------

VIN13_Transformant_1 ------------------------------------------------------------

VIN13_CONTROL ------------------------------------------------------------

BY4741_Transformant_9 ------------------------------------------------------------

FIT2_FLANKING_AND_CDS AGCATCTTGGGTGACAGTCTTAGTGTAGACATGACCGTTCTTGGTGGCAGTGATGGTGGT

VIN13_Transformant_7 ------------------------------------------------------------

VIN13_Transformant_6 ---

VIN13_Transformant_2 ---

VIN13_Transformant_1 ---

VIN13_CONTROL ---

BY4741_Transformant_9 ---

FIT2_FLANKING_AND_CDS GGT

VIN13_Transformant_7 ---
